# The Potential of MLN3651 in Combination with Selumetinib as a Treatment for Merlin-Deficient Meningioma

**DOI:** 10.3390/cancers12071744

**Published:** 2020-06-30

**Authors:** Jade Lyons Rimmer, Emanuela Ercolano, Daniele Baiz, Mahindra Makhija, Allison Berger, Todd Sells, Steve Stroud, David Hilton, Claire L. Adams, C Oliver Hanemann

**Affiliations:** 1 Peninsula Schools of Medicine and Dentistry, Institute of Translational and Stratified Medicine, Plymouth University, Plymouth PL68BU, UK; jade.lyonsrimmer@plymouth.ac.uk (J.L.R.); emanuela.ercolano@plymouth.ac.uk (E.E.); danbaiz73@gmail.com (D.B.); claire.adams@plymouth.ac.uk (C.L.A.); 2 Takeda International UK, 1 Kingdom Street, London W2 6BD, UK; mahindra.makhija@takeda.com; 3 Millennium Pharmaceuticals, Inc. a Wholly Owned Subsidiary of Takeda Pharmaceutical Company Limited, Cambridge, MA 02139, USA; Allison.Berger@Takeda.com (A.B.); toddsells01@gmail.com (T.S.); steve.stroud@takeda.com (S.S.); 4 Department of Histopathology, University Hospitals Plymouth NHS Trust, Plymouth, Devon PL6 8DH, UK; davidhilton@nhs.net

**Keywords:** Merlin, meningioma, CRL4-DCAF1, KSR1, Raf/MEK/ERK

## Abstract

Meningioma is the most common primary intracranial tumour, and surgical resection is the main therapeutic option. Merlin is a tumour suppressor protein that is frequently mutated in meningioma. The activity of the E3 ubiquitin ligase complex, CRL4-DCAF1, and the Raf/MEK/ERK scaffold protein Kinase suppressor of Ras 1 (KSR1) are upregulated in Merlin-deficient tumours, which drives tumour growth. Identifying small molecules that inhibit these key pathways may provide an effective treatment option for patients with meningioma. We used meningioma tissue and primary cells derived from meningioma tumours to investigate the expression of DDB1 and Cullin 4-associated factor 1 (DCAF1) and KSR1, and confirmed these proteins were overexpressed. We then used primary cells to assess the therapeutic potential of MLN3651, a neddylation inhibitor which impacts the activity of the CRL family of E3 ubiquitin ligases and the MAPK/ERK kinase (MEK1/2) inhibitor selumetinib. MLN3651 treatment reduced proliferation and activated apoptosis, whilst increasing Raf/MEK/ERK pathway activation. The combination of MLN3651 and the MEK1/2 inhibitor selumetinib prevented the increase in Raf/MEK/ERK activity, and had an additive effect compared with either treatment alone. Therefore, the combined targeting of CRL4-DCAF1 and Raf/MEK/ERK activity represents an attractive novel strategy in the treatment of Merlin-deficient meningioma.

## 1. Introduction

Meningioma is the most common intracranial tumour that arises from the arachnoid and dural border cells of the leptomeninges surrounding the brain and spinal cord [1,2,3]. Meningioma, which are graded I–III depending on aggressiveness, cause symptoms such as headaches and/or focal neurological deficits [4]. The primary treatment for meningioma is surgery followed by radiotherapy for high-grade tumours. However, complete resection is not always possible, and therefore recurrence is more likely, particularly in patients with higher grade tumours [5]. Currently, there is no effective pharmacological intervention for meningioma. Merlin-deficient meningioma arise when both alleles of the Neurofibromin 2 gene (*NF2*) are mutated, which underlie the genetic condition neurofibromatosis type 2 (NF2), and occur sporadically in more than 50% of meningioma [6,7]. Other tumours commonly associated with Merlin loss or inactivation include schwannomas, ependymomas, mesotheliomas, and a variety of other cancers [8]. Merlin is a tumour suppressor protein, that acts as a contact-dependent inhibitor of growth by suppressing or activating cell signalling pathways, and preventing cytoskeletal reorganisation [9]. Merlin inhibits growth by regulating the cell membrane expression of several receptor tyrosine kinases, including platelet-derived growth factor receptor (PDGFR), erythroblastic oncogene B homolog 2 (ERBB2) and erythroblastic oncogene B homolog 3 (ERBB3), preventing activation of downstream growth pathways such as PI3K/AKT and Raf/MEK/ERK [10,11,12]. Therefore, Merlin loss leads to increased cell membrane recruitment of receptor tyrosine kinases, and aberrant activation of these pathways. Merlin also acts in the nucleus during the early G1 cell cycle phase, and competes with CRL4 to bind the carboxy-terminal tail of DDB1 and Cullin 4-associated factor 1 (DCAF1) to prevent ubiquitin substrate recruitment [13,14]. Thus, in the absence of Merlin, CRL4-DCAF1 activity (comprising Cullin 4A/B, the adaptor protein DNA damage-binding protein 1 (DDB1), and DCAF1 as the substrate receptor protein) is enhanced [12,15,16]. CRL4-DCAF1 ubiquitinates Large tumor suppressor kinase 1/2 (LATS1/2), and prevents Yes-associated protein (YAP) phosphorylation, thus inhibiting the Hippo tumour suppressor pathway and supporting tumour growth [13]. The neddylation inhibitor, pevonedistat (MLN4924), has been shown to inhibit CRL4-DCAF1 activity in Merlin-mutant mesothelioma and schwannoma, in order to activate the Hippo pathway and suppress growth [17]. The CRL4-DCAF1 complex has also been shown to interact with Kinase suppressor of Ras 1 (KSR1), a scaffold protein of the Raf/MEK/ERK pathway [18,19]. Interestingly, KSR1 has a key role in schwannoma pathogenesis; KSR1 knockdown in primary schwannoma led to reduced proliferation, increased apoptosis and altered cell morphology [18]. Here, we aim to further characterise the expression of DCAF1 and KSR1 in Merlin-deficient grade I meningioma (herein referred to as meningioma), and assess the therapeutic potential of targeting these proteins/pathways using the neddylation inhibitor MLN3651, and the MEK1/2 inhibitor selumetinib [20]. We used the neddylation inhibitor MLN3651, which is structurally distinct from the first-in-class neddylation inhibitor pevonedistat, to decrease the E3 ligase activity of CRLs, including CRL4-DCAF1, in primary meningioma cells. Primary human meningioma cells were the best model for investigating our hypothesis, not only because these cells do not grow easily in patient-derived xenografts, but they also allowed us to easily evaluate response in Merlin-positive, Merlin-negative, WHO grade I and WHO grade II tumours.

## 2. Results

### 2.1. CRL4-DCAF1 and KSR1 Are Overexpressed in Meningioma Compared to Normal Cells

**Figure 1:**
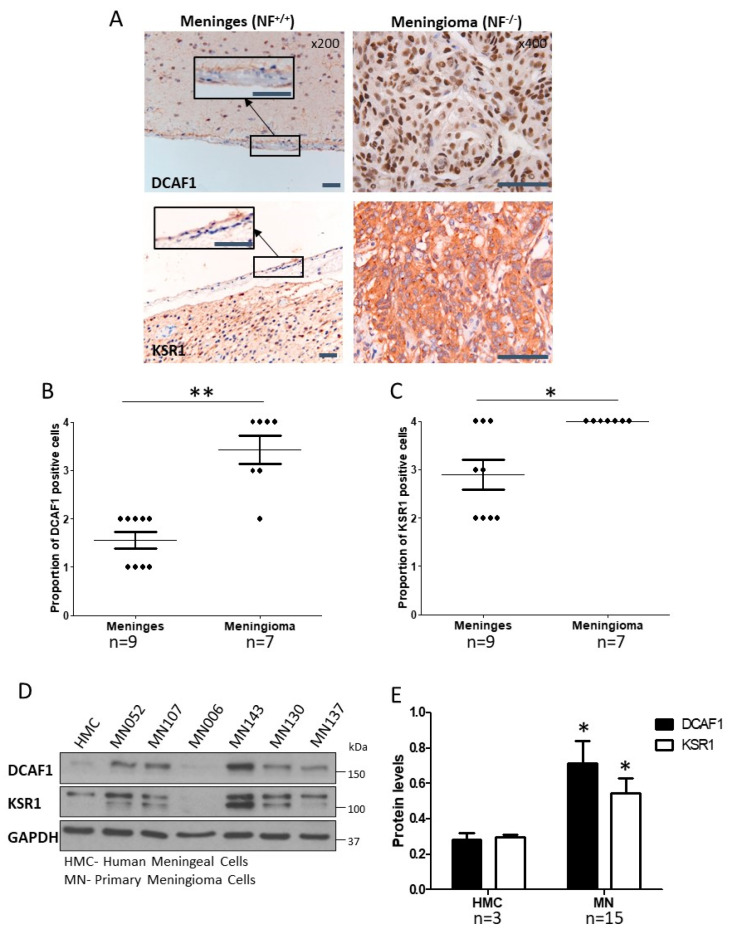
DDB1 and Cullin 4-associated factor 1 (DCAF1) and Kinase suppressor of Ras 1 (KSR1) are overexpressed in Merlin-deficient grade I meningioma, compared to normal cells. (**A**) Representative immunohistochemical images of normal meninges (200× with 400× insert) (*n* = 9) and meningioma (400×) (*n* = 7) tissue, stained with DCAF1 and KSR1 antibodies and counterstained with haematoxylin. In meningioma, DCAF1 expression was mostly nuclear with some diffuse cytoplasmic expression, whilst KSR1 expression was diffuse and granular in the cytoplasm, with some strong paranuclear expression. Scale bar—50 µM; (**B**) and (**C**) Proportions of DCAF1 and KSR1 positive cells in nine normal meninges and seven meningioma tissues are plotted, where a score of 1 was less than 25% of cells expressing the protein, a score of 2 was between 25% and 50%, a score of 3 was between 51% and 75% and a score of 4 was more than 75%. ** *p* < 0.01, * *p* < 0.05; (**D**) Representative Western blot showing DCAF1 and KSR1 expression in human meningeal cells (HMC; *n* = 3) and meningioma cells (MN; *n* = 14); (**E**) Mean DCAF1 and KSR1 expression and standard error of the mean (SEM) in HMC and MN normalised to the loading control, GAPDH. * *p* < 0.05, ** *p* < 0.01. The whole western blot image please find in Figure S8.

We investigated both DCAF1 and KSR1 expression in meningioma. DCAF1 was expressed in the nucleus, and significantly increased in meningioma tissue, compared to the arachnoid layer of normal meninges by immunohistochemistry [*p* < 0.01; Figure 1A,B (shown in insert); Figure S1A,B]. We also showed that DCAF1 expression was increased in Merlin-deficient schwannoma tissue compared to normal nerve, suggesting that the loss of Merlin leads to the accumulation of DCAF1 protein (Figure S2A,B). We then confirmed that KSR1 was significantly increased in meningioma tissue (*p* < 0.05; Figure 1A,C). Primary cells were derived from grade I meningioma tissue, and the loss of Merlin protein was confirmed by Western blot (a representative blot is shown in Figure S2C). DCAF1 and KSR1 were significantly overexpressed in meningioma cells compared to normal human meningeal cells (HMC) (*p* < 0.05 and *p* < 0.01, respectively; Figure 1D,E). There was variation in DCAF1 and KSR1 expression between different meningioma samples (Figure 1D). However, there was a positive correlation between DCAF1 and KSR1 expression within individual samples (Figure S2D). 

### 2.2. Inhibition of CRL4-DCAF1 Neddylation by MLN3651 Induces Apoptosis and Inhibits Proliferation to Reduce Cell Viability in Meningioma 

**Figure 2:**
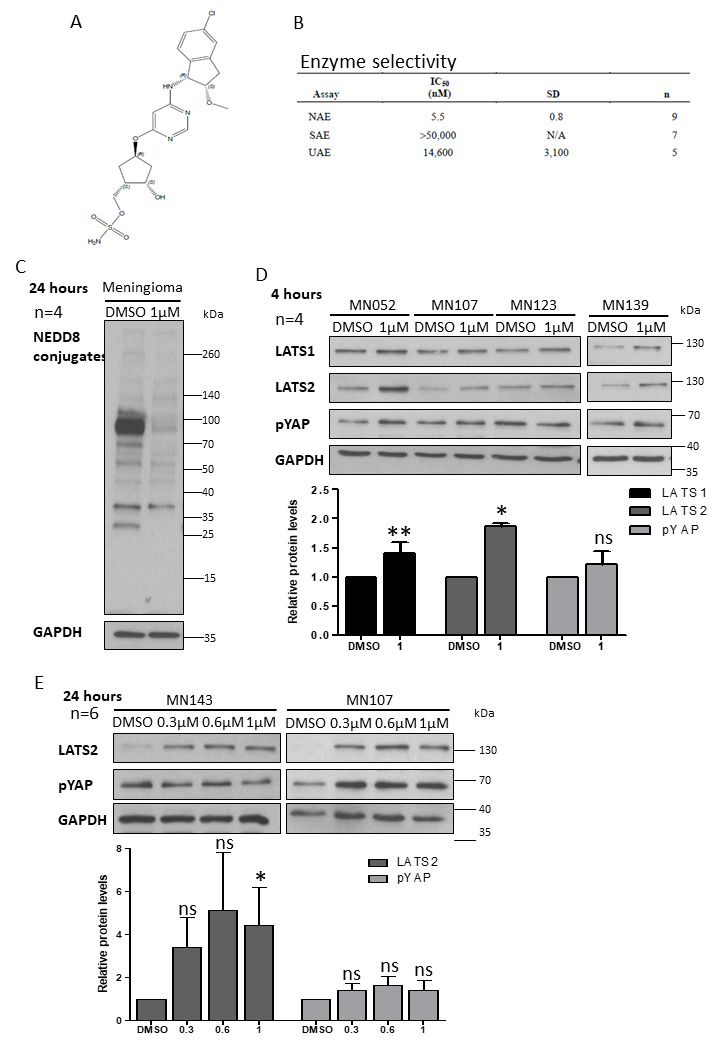
Inhibition of CRL4-DCAF1 neddylation by MLN3651. (**A**) MLN3651 chemical structure; (**B**) The table shows the neddylation activating enzyme (NAE) IC_50_ values, determined using the Ubc12-NEDD8 Homogeneous Time Resolved Fluorescence (HTRF) thioester assay, and the IC_50_ values for related E1enzymes—SUMO activating enzyme (SAE) and ubiquitin activating enzyme (UAE)—were determined using the Ubc9-Sumo1 or Ubc2-Ub–HTRF thioester assays, respectively [21,22]; (**C**) Representative Western blot of four primary meningioma (MN)-treated with Dimethyl sulfoxide (DMSO) or 1 µM MLN3651 for 24 h, and probed for NEDD8-conjugates and Glyceraldehyde 3-phosphate dehydrogenase (GAPDH); (**D**) Western blots of meningioma cells treated with 1 µM MLN3651 for 4 h and probed for LATS1, LATS2, pYAP and GAPDH. The graph shows the mean protein expression with standard error of the mean (SEM) in MLN3651-treated Meningioma (MN) cells normalised to the loading control, GAPDH and relative to Scramble, ns—not significant. ** *p* < 0.01, * *p* < 0.05; (**E**) Representative Western blots of six primary meningioma treated with DMSO, 0.3 µM, 0.6 µM or 1 µM MLN3651 for 24 h, and probed for LATS2, pYAP and GAPDH. MLN3651 led to an increase in pYAP in three out of six meningioma. The graph shows the mean protein expression with standard error of the mean (SEM) in MLN3651-treated MN cells normalised to the loading control, GAPDH and relative to Scramble. ns—not significant, * *p* < 0.05. The whole western blot image please find in Figure S8.

The structure of MLN3651 and its high selectivity for NEDD8-activating enzyme (NAE), compared to Small Ubiquitin-like Modifier (SUMO)-activating enzyme and ubiquitin-activating enzyme, are detailed in Figure 2A,B [21,22]. We treated meningioma cells with 1 µM MLN3651 for 24 h, and showed a clear reduction in NEDD8-conjugates, confirming that neddylation was inhibited (Figure 2C). Then, we analysed Hippo pathway activation, showing that MLN3651 increased LATS1/2 expression in meningioma cells after 4 hours, suggesting CRL4-DCAF1 inhibition. However, the levels of phosphorylated YAP remained unchanged (Figure 2D). We then treated meningioma cells with increasing concentrations of MLN3651 for 24 h, to determine if the Hippo pathway would be activated at a later time point. Whilst LATS2 remained elevated in response to MLN3651 treatment, pYAP expression was only enhanced in some meningioma (3/6), suggesting an efficient feedback pathway that inhibits YAP phosphorylation (Figure 2E). We confirmed increased LATS2 expression and sustained Hippo pathway activation (pYAP) in Merlin-deficient schwannoma after MLN3651 treatment for 72 h, suggesting that MLN3651 inhibits CRL4-DCAF1 activity in Merlin-deficient cells (Figure S3A).

**Figure 3:**
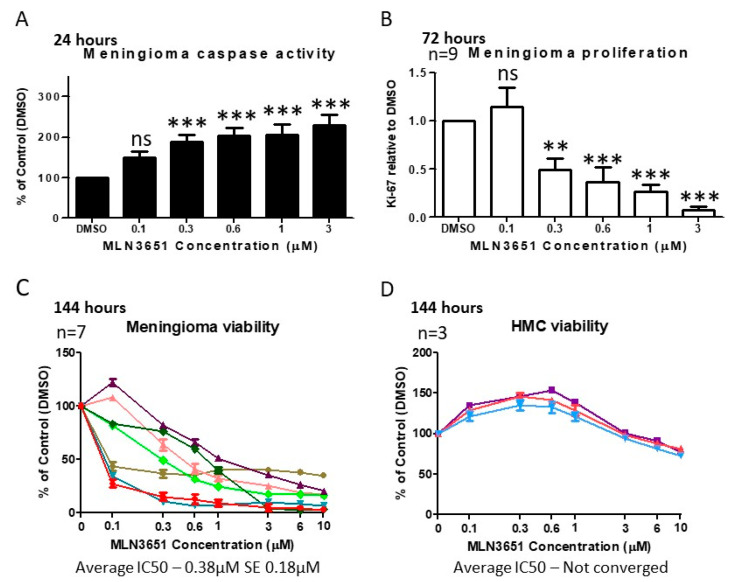
MLN3651 induces apoptosis and inhibits proliferation to reduce the cell viability of meningioma. (**A**) Meningioma cells were treated with DMSO (*n* = 10), 0.1 µM (*n* = 4), 0.3 µM (*n* = 10), 0.6 µM (*n* = 10), 1 µM (*n* = 10) or 3 µM (*n* = 6) MLN3651, and Caspase3/7 activity was assessed after 24 h. Caspase 3/7 activity was significantly higher in MLN3651-treated cells compared with DMSO-treated cells, *** *p* < 0.001; (**B**) Meningioma cells treated with DMSO, 0.1 µM, 0.3 µM, 0.6 µM, 1 µM or 3 µM MLN3651 for 72 h, and stained with Ki-67 antibody and 4′,6-diamidino-2-phenylindole (DAPI). MLN3651 significantly reduced proliferation of meningioma compared with DMSO, ** *p* < 0.01, *** *p* < 0.001 (*n* = 9); (**C**) Meningioma cells were treated with increasing doses of MLN3651 and cell viability was assessed after 144 h (*n* = 7); (**D**) Human meningeal cells (HMC), a normal meningeal control cell line, were treated with MLN3651 and cell viability was assessed at 144 h (g) (*n* = 3).

To assess the impact of MLN3651 on apoptosis, we measured cleaved caspase 3/7 in response to increasing MLN3651 concentrations. In meningioma cells, MLN3651 induced apoptosis, as evidenced by a significant increase in cleaved caspase 3/7 in the treated cells compared to the control cells after 24 h (*p* < 0.001; Figure 3A). In addition, we identified a dose-dependent reduction in the proliferation (as measured by the proportion of Ki-67 positive cells) of Merlin-deficient grade I meningioma cells after 72 h of MLN3651 treatment (Figure 3B). We then determined cell viability using the CellTiter-Glo^®^ Luminescent Cell Viability Assay, which measures ATP and is proportional to the number of metabolically active cells. In this experiment, we used the viability assay to determine differences in cell number due to the reduction of proliferation and increased apoptosis at 72 and 144 h post-treatment with MLN3651. After 72 h, it was evident that Merlin-deficient grade I meningioma cells displayed either a responsive or less-responsive treatment profile. The less-responsive meningioma cells had a much higher average IC_50_ of 12.81 µM, compared to responsive tumours, which had an average IC_50_ of 1.31 µM (Figure S4A,B). However, following 144 hours of incubation, all meningioma showed a responsive profile with an average IC_50_ of 0.38 µM, suggesting that MLN3651 has a maximum effect at different time points in individual meningioma (Figure 3C). We also treated HMC cells with MLN3651 for 72 h, and demonstrated a minimal response to treatment comparable to the less-responsive meningioma (Figure S4C). Importantly, after 144 h there was only a slight reduction in viability at higher concentrations and an IC_50_ > 10 µM, suggesting that these cells are not sensitive to MLN3651 treatment, even for long periods (Figure 3D). Furthermore, Merlin-positive grade I meningioma cells showed a similar profile to the less-responsive Merlin-deficient meningioma cell samples, with an average IC_50_ of 10.12 µM (Figure S4D). We also treated available grade II meningioma, and confirmed that Merlin-deficient meningioma were more sensitive to MLN3651 than Merlin-positive meningioma after 72 h treatment (Figure S5A). Interestingly, Merlin-positive grade II meningioma cells responded to MLN3651 treatment after 144 h (IC_50_ < 3 µM), however, Merlin-deficient grade II meningioma cells remained more sensitive (IC_50_ < 1 µM) (Figure S5B).

### 2.3. MLN3651 and DCAF1 Knockdown Effect on the Raf/MEK/ERK Pathway in Meningioma 

**Figure 4:**
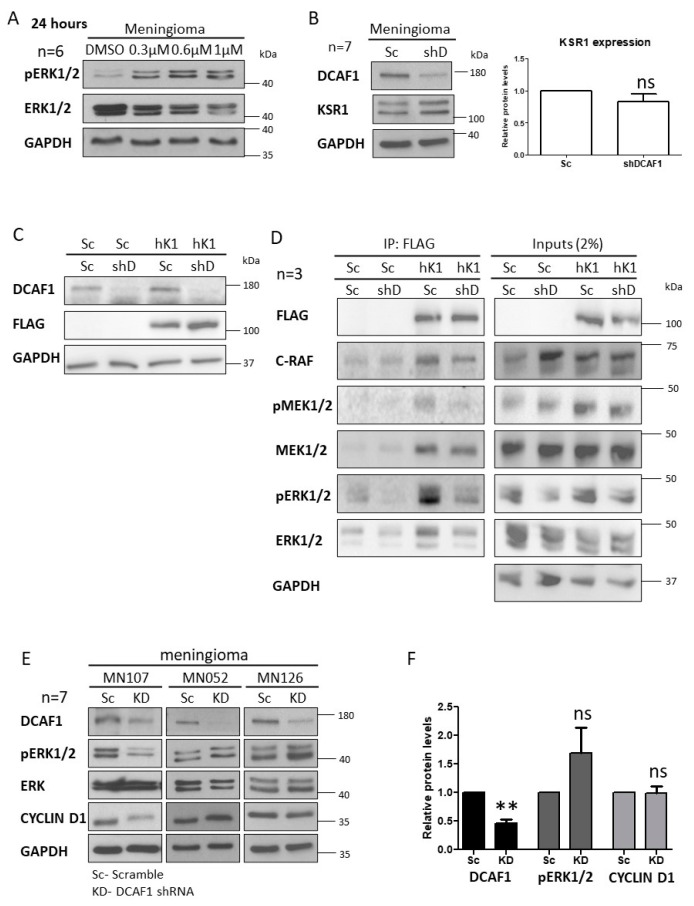
Raf/MEK/ERK activity is enhanced after MLN3651 treatment or DCAF1 knockdown in meningioma. (**A**) Western blot analysis of three primary meningioma (MN) treated with DMSO, 0.3 µM, 0.6 µM or 1 µM MLN3651 for 24 hours, and probed for pERK1/2, ERK1/2 and GAPDH (loading control; *n* = 6); (**B**) Representative Western blot of DCAF1 and KSR1 expression after DCAF1 knockdown. Scramble (Sc) or DCAF1 (KD) lentivirus was added to cells followed by seven days of puromycin selection before cell lysis (*n* = 7). The graph shows the mean KSR1 expression with standard error of the mean (SEM) in shDCAF1-treated MN cells normalised to the loading control, GAPDH and relative to Scramble, ns—not significant; (**C**) DCAF1 expression was targeted with a shRNA lentiviral plasmid in HEK293T cells, and then human KSR1 (hK1) with a FLAG tag was overexpressed; (**D**) Western blot shows immunoprecipitated FLAG complexes and input lysates probed with FLAG, C-RAF, pMEK1/2, MEK1/2, pERK1/2. ERK1/2 and GAPDH, representative of three repeats. FLAG tag interacting proteins were immunoprecipitated from Scramble (Sc)/Sc and Sc/DCAF1 knockdown (shD) lysates as a negative control. Input lysates show endogenous expression in lysates prior to the immunoprecipitation (2% volume); (**E**) Western blots of DCAF1, pERK1/2, ERK1/2 and CYCLIN D1 expression after Scramble (Sc) or DCAF1 knockdown (KD) in three meningioma (MN) representative of seven replicates; (**F**) Mean DCAF1, pERK1/2 and CYCLIN D1 expression and SEM in shDCAF1-treated MN cells normalised to the loading control, GAPDH and relative to Scramble. (** *p* < 0.01, ns: not significant, *p* > 0.05). The whole western blot image please find in Figure S8.

MLN3651 treatment of Merlin-deficient grade I meningioma for 24 hours led to an increase in pERK1/2 expression, suggesting that Raf/MEK/ERK is activated in response to MLN3651 (Figure 4A). CRL4-DCAF1 interacts with KSR1, therefore we aimed to determine if DCAF1 could regulate KSR1 expression or activity, in order to explain why MLN3651 activates Raf/MEK/ERK [18]. We confirmed that DCAF1 knockdown in meningioma did not alter KSR1 protein levels (Figure 4B,C). To determine if CRL4-DCAF regulates the activity of KSR1, we knocked down DCAF1 expression and overexpressed human KSR1 with a FLAG tag in HEK293T cells (Figure 4C). FLAG-KSR1 was immunoprecipitated, and its physical interaction with components of the Raf/MEK/ERK pathway determined. DCAF1 knockdown led to a reduction of the amount of pMEK1/2 and pERK1/2 associated with KSR1, suggesting that DCAF1 facilitates KSR1-dependent Raf/MEK/ERK activation (Figure 4D). However, this novel function of CRL4-DCAF1 does not explain why MLN3651 enhances Raf/MEK/ERK activity in meningioma. We explored if the reduced activation of the Raf/MEK/ERK pathway was mirrored in meningioma following DCAF1 knockdown. We found that pERK1/2 expression was decreased in some samples and increased in others (Figure 4E,F). Overall, these results suggest that Raf/MEK/ERK signalling is not dependent on the CRL4-DCAF1-mediated activation of KSR1 in meningioma. Interestingly, DCAF1 knockdown in primary Merlin-deficient schwannoma samples led to a significant decrease in pERK activity, suggesting that Raf/MEK/ERK activity is more dependent on KSR1 activation in schwannoma than in meningioma (Figure S6A,B).

### 2.4. Simultaneous Inhibition of CRL4-DCAF1 and MEK1/2 Leads to an Enhanced Therapeutic Response in Meningioma

**Figure 5:**
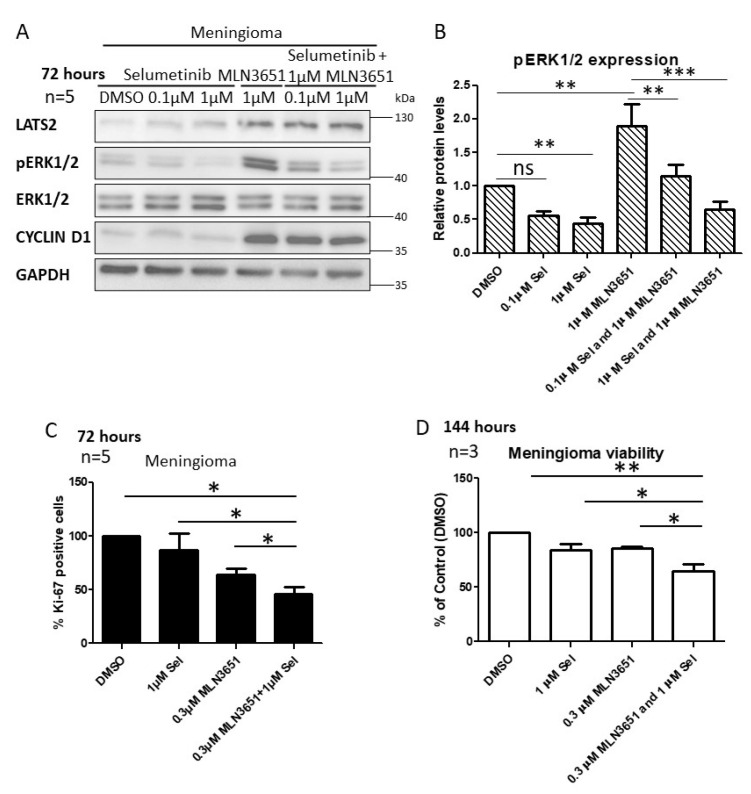
Simultaneous inhibition of CRL4-DCAF1 and MEK1/2 has an enhanced effect on proliferation and cell viability of meningioma. (**A**) Western blot of primary meningioma cells treated with MLN3651 and/or selumetinib (Sel) for 72 hours, and probed for LATS2, pERK1/2, ERK1/2, CYCLIN D1 and GAPDH, representative of five replicates; (**B**) The graph shows the mean pERK1/2 expression with standard error of the mean (SEM), in combination with treated meningioma cells from (A) normalised to both total ERK1/2 and the loading control, Glyceraldehyde 3-phosphate dehydrogenase(GAPDH), and relative to DMSO, ns: not significant, ** *p* < 0.01, *** *p* < 0.0001; (**C**) Meningioma cells were treated with DMSO, 1 µM Sel, 0.3 µM MLN3651 or 1 µM Sel and 0.3 µM MLN3651 for 72 h and stained with Ki-67 antibody and DAPI. At least three images each of five replicates were quantified to calculate the percentage of Ki-67 positive cells. Combination of Sel and MLN3651 significantly reduced proliferation compared with either treatment alone, * *p* < 0.05; (**D**) Meningioma cells were treated with DMSO, 1 µM Sel, 0.3 µM MLN3651 or 1 µM Sel and 0.3 µM MLN3651 for 144 h, with three technical replicates, and viability was calculated comparative to the DMSO control, * *p* < 0.05, ** *p* < 0.01. The whole western blot image please find in Figure S8.

Based on our results, demonstrating Raf/MEK/ERK activation in response to MLN3651, we hypothesised that a combination of MLN3651 and the MEK1/2 inhibitor, selumetinib, may have therapeutic potential in Merlin-deficient meningioma. Meningioma cells were treated for 72 h with MLN3651, selumetinib or a combination of both compounds. MLN3651 treatment in combination with selumetinib decreased pERK1/2 expression, compared with MLN3651 alone but not compared with the vehicle-treated control after 72 h in meningioma (Figure 5A,B). We then tested the effect of 1 µM MLN3651 and 1 µM selumetinib on proliferation after 72 h, and showed that the combination significantly reduced proliferation, compared to 1 µM selumetinib alone but not compared to 1 µM MLN3651 alone (Figure S7A). Furthermore, we tested the combination of 0.3 µM MLN3651 and 1 µM selumetinib, and showed that this significantly reduced proliferation compared with either inhibitor alone after 72 h, demonstrating the increased efficacy of the drug combination (*p* < 0.05; Figure 5C). In addition, the viability of meningioma cells was significantly decreased by the combination treatment of selumetinib and MLN3651 for 144 h (*p* < 0.05; Figure 5D).

## 3. Discussion

**Figure 6:**
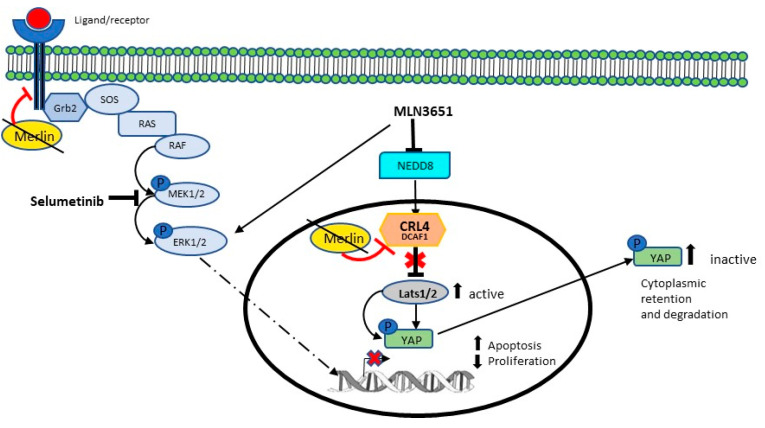
Inhibition of CRL4-DCAF1 and Raf/MEK/ERK signalling in Merlin-deficient meningioma. When Merlin is lost, there is an increased accumulation of growth factor receptors at the cell membrane leading to activation of downstream signalling [33]. Activation of RAF/MEK/ERK leads to increased gene transcription of the genes involved in proliferation and downregulation of pro-apoptotic proteins. CRL4-DCAF1 is hyper-activated when Merlin is lost and inhibits LATS1/2, in the nucleus, therefore releasing the LATS1/2-dependent inhibition of the YAP function, allowing YAP to regulate gene transcription [13]. MLN3651 inhibits neddylation and inhibits the activity of the CRL family of E3 ubiquitin ligases, including CRL4-DCAF1, as well as activating Raf/MEK/ERK. The MEK1/2 inhibitor selumetinib inhibits Raf/MEK/ERK activation when combined with MLN3651, and has an additive effect on the inhibition of proliferation and activation of apoptosis in Merlin-deficient meningioma.

We provide evidence showing that combination treatment, with a neddylation inhibitor and a MEK1/2 inhibitor, is effective in targeting two upregulated key pathways in meningioma (summarised in Figure 6). Merlin inhibits CRL4-DCAF1 by directly binding to the DCAF1 substrate recruitment domain, and therefore, Merlin-deficient tumours have increased CRL4-DCAF1 activity [13]. We show that increased DCAF1 protein expression further increases CRL4-DCAF1 activity in Merlin-deficient meningioma and schwannoma. KSR1 expression was shown to be increased in Merlin-deficient schwannoma, and is a regulator of proliferation, apoptosis, morphology and adhesion [18]. We show that KSR1 is also increased in Merlin-deficient grade I meningioma, suggesting that KSR1 also has a role in meningioma tumourigenic signalling. In vitro, we observed variability in the DCAF1 and KSR1 expression in cultured primary meningioma cell samples. Angus et al., 2018, also reported variability in EphA2 and Src protein expression in primary Merlin-deficient meningioma, demonstrating the heterogeneity in the expression levels of cultured primary meningioma samples [23]. In addition, we determined Merlin status by the absence of the full length protein via Western blot, and we did not characterise meningioma samples based on the type of Merlin mutation. Therefore, we cannot exclude that each Merlin mutation type has a differential effect on the expression of DCAF1 and KSR1. 

Merlin has been shown to modulate the Hippo pathway through inhibition of CRL4-DCAF1-dependent ubiquitination of LATS1/2, leading to YAP phosphorylation and cytoplasmic retention/degradation [13]. To inhibit CRL4-DCAF1, we treated meningioma cells with MLN3651, and showed that NEDD8-conjugates were significantly reduced with treatment. Furthermore, we showed that MLN3651 led to an increase in LATS1/2 protein, suggesting that the CRL4-DCAF1 ubiquitination of LATS1/2 is inhibited. Consistent with this, in vitro treatment of a mouse schwannoma cell line (FC-1801), with the first in class neddylation inhibitor pevonedistat, reduced LATS1/2 ubiquitination and increased YAP phosphorylation [17]. Whilst we did not see a consistent increase in pYAP at the time points tested in meningioma, we were able to show that MLN3651 treatment of Merlin-deficient schwannoma increased both LATS2 and pYAP, demonstrating that MLN3651 is able to activate the Hippo pathway. Similarly, Cooper et al., 2017, noted a relapse of YAP-inhibitory phosphorylation after 36 hours in the Meso-33 Merlin-mutant mesothelioma cell line, which may explain why we did not see a consistent increase in pYAP expression in Merlin-deficient meningioma [17]. MLN3651 treatment activated apoptosis and inhibited the proliferation of meningioma, which led to a significant reduction in cell viability consistent with previous reports using pevonedistat in other Merlin-deficient models [17]. It is also possible that MLN3651 activated apoptosis and inhibited proliferation in a DCAF1-independent manner, as MLN3651 reduces NEDD8-conjugation of many proteins. However, we showed that normal human meningeal cells were relatively resistant to MLN3651 when treated for 144 h, suggesting that neddylation inhibition is an attractive therapeutic strategy for Merlin-deficient meningioma. 

We report the increased sensitivity of Merlin-deficient grade I and II meningioma cells to MLN3651 treatment, compared with Merlin-positive meningioma cells. Importantly, higher grade and more aggressive meningiomas show the greatest Merlin loss, and successful treatment of these tumours represents an important unmet need [24,25,26]. Cooper et al. also reported that pevonedistat preferentially inhibits the proliferation of Merlin-mutant mesothelioma and schwannoma cells, compared to Merlin-positive cells [17]. In fact, the half-maximal growth inhibitory concentration was 10 times higher in the Merlin-expressing FH-912 Schwann cell line than the Merlin-deficient FC-1801 schwannoma cell line [17]. The increased activity and expression of CRL4-DCAF1 in Merlin-deficient meningioma, compared to Merlin-positive control cells, could explain why they have increased sensitivity to MLN3651, and therefore, the expression of both CRL4-DCAF1 and KSR1 should be explored further in Merlin-positive meningioma. The increased sensitivity of Merlin-deficient tumours to MLN3651 also suggests that it could be effective in other tumours in which there are inactivating Merlin mutations, such as schwannoma, ependymoma, mesothelioma, glioma and hepatocellular carcinoma [27,28,29]. 

The Raf/MEK/ERK pathway contributes to tumour growth in Merlin-deficient tumours, and KSR1 is upregulated in meningioma. Therefore, we analysed Raf/MEK/ERK activity after MLN3651 treatment [18]. Interestingly, MLN3651 enhanced pERK protein levels, which could be in a CRL4-DCAF1-dependent manner. Previously, Zhou et al. and Dougherty et al. showed that CRL4-DCAF1 and KSR1 interact in HEK293T cells, and DCAF1 knockdown in schwannoma did not lead to changes in the protein levels of KSR1 [18,19]. We show that DCAF1 knockdown did not change the levels of KSR1 protein in meningioma, and therefore DCAF1 is unlikely to target KSR1 for proteasomal degradation. Zhou et al. suggested that active Merlin inhibits KSR1’s association with c-Raf, and reduces pMEK1/2 [18]. We show here that DCAF1 knockdown reduced KSR1 association with binding partners of the Raf/MEK/ERK pathway in HEK293T cells, suggesting that this inhibition occurs in a CRL4-DCAF1-dependent manner and that DCAF1 enhances KSR1’s scaffold function. This novel mechanism could explain why DCAF1 knockdown leads to reduced pERK1/2 levels in Merlin-deficient schwannoma, and why combined shRNA knockdown of DCAF1 and KSR1 reduced the proliferation of schwannoma cells [18]. However, a reduction of pERK1/2 in response to DCAF1 knockdown was only observed in some meningioma samples, providing a rationale for combination therapy targeting DCAF1 and Raf/MEK/ERK. In addition, pERK1/2 activity was increased in MLN3651-treated meningioma. There could be a feedback mechanism in meningioma that permits pERK1/2 activation independent of KSR1 in DCAF1-depleted cells. Indeed, Angus et al. showed increased pSFK and total Src in response to MEK inhibitors, supporting the role of Raf/MEK/ERK adaptive signalling in meningioma [23]. This should be explored further in order to identify additional pathways that could be targeted. In respect to MLN3651 treatment, it is also important to understand that all E3 ubiquitin ligase proteins that are activated by NEDD8, as well as other proteins regulated by neddylation, are targeted. Therefore, the increase in pERK1/2 observed could also be CRL4-DCAF1-independent. This observation of activated Raf/MEK/ERK in response to MLN3651 in meningioma provides a further rationale for targeting the Raf/MEK/ERK pathway in combination with MLN3651.

Based on our results, we hypothesised that combining MLN3651 and selumetinib would have a beneficial effect, by targeting two mechanisms of Merlin-deficient tumourigenesis simultaneously. Interestingly, Yang et al. showed that combining pevonedistat and sorafenib had additive effects for the inhibition of hepatocellular carcinoma proliferation and migration, as well as inducing apoptosis [30]. We chose to combine MLN3651 with selumetinb, because selumetinib has been tested in a variety of clinical trials, either alone or in combination, with tolerable side effects [31,32]. In meningioma, combination of MLN3651 and selumetinib inhibited Raf/MEK/ERK activation induced by MLN3651, whilst maintaining CRL4-DCAF1 inhibition through increased LATS2. In addition, 0.3 µM MLN3651 and selumetinib significantly decreased proliferation and cell viability, compared with either treatment alone, suggesting an additive or synergistic effect, which should be explored further.

## 4. Materials and Methods 

### 4.1. Human Tissue

Primary meningioma were obtained from consented individuals following the ethical guidelines included in the ‘Investigation into the expression of signalling molecules in human brain tumour samples (Research Ethics Committee (REC) number 6/Q2103/123)’ and the ‘Identifying and validating molecular targets in low grade brain tumours (REC number 14/SW/0119) study’. Primary tumours were mechanically disrupted after overnight incubation with a digestion medium. Meningioma medium consisted of DMEM, 10% FBS, 100 U/mL penicillin/streptomycin and 20 U/mL collagenase type III (Sigma-Aldrich, Dorset, UK). The digested sample was collected and centrifuged, the cells were then cultured in meningioma medium [DMEM, 10% FBS, 100 U/mL penicillin/streptomycin, 2 mM L-glutamine (Gibco) and 1% v/v D(+) glucose solution (Sigma)]. Merlin protein expression was determined by Western blot and only samples with no expression were used in subsequent Merlin-deficient experiments (Figure S1A). Human meningeal cells (HMC) (Sciencell, Carlsbad, CA, USA) and 293T cells were cultured as directed by the manufacturer. 

### 4.2. Co-Immunoprecipitation 

FLAG-KSR1 was immunoprecipitated from 1 mg HEK293T using ANTI-FLAG^®^ M2 Affinity Gel (Sigma-Aldrich) following the manufacturer’s instructions.

### 4.3. Drug Treatments 

MLN3651, a NEDD8-activating enzyme (NAE) inhibitor, was provided by Millennium Pharmaceuticals, Inc., Cambridge, MA, a wholly owned subsidiary of Takeda Pharmaceutical Company Limited, at a concentration of 10 mM in Dimethyl sulphoxide (DMSO). The MEK1/2 inhibitor selumetinib (Selleckchem, Houston, TX, USA) was resuspended in DMSO. Final drug concentrations were made in phenol-free cell culture medium to achieve a final concentration of 0.1% DMSO. Primary cells were plated in 96-well culture plates (Greiner Bio-One; #655088) at 3000 cells per well. After 24 h, the medium was removed and replaced with varying concentrations of MLN3651 or selumetinib. Cells were incubated for 24, 72 or 144 h and cell viability or apoptosis determined using the Celltiter-Glo^®^ Luminescent Cell Viability Assay (Promega) or Caspase-Glo^®^ 3/7 Assay (Promega, Madison, WI, USA), respectively. ANOVA with Tukey’s Multiple Comparison Post Test was used to test statistical significance between treated cells and DMSO control (*p* < 0.05). IC_50_ was determined using Graphpad Prism 5 with automatic outlier elimination. 

### 4.4. Immunocytochemistry 

Immunocytochemistry was performed using 8-well chamber slides (Nunc™ Lab-Tek™ Chamber Slide System, Thermo Fisher Scientific, Waltham, MA, USA. Briefly, 5000 or 10,000 cells (meningioma and schwannoma, respectively) were plated in each well, and left to grow overnight before adding drug treatments. After the drug treatment, the cells were fixed with 4% paraformaldehyde (Thermo Fisher Scientific)/PBS. Cells were permeabilised with 0.2% Triton X-100 for 5 min; blocking was performed with 10% v/v goat serum (Abcam, Cambridge, UK) in 1% w/v bovine serum albumin (BSA) (Thermo Fisher Scientific)/PBS for 1 h. Cells were incubated overnight with Ki-67 (1:200) (DAKO) in 1% BSA/PBS and then Goat anti-Mouse IgG (H+L) Cross-Adsorbed Secondary Antibody, Alexa Fluor 594 (Thermo Fisher Scientific) (1:500) in 1% BSA/PBS for 1 h at room temperature. Finally, 4’,6-diamidino-2-phenylindole (DAPI) nuclear stain was added (1:500) before imaging with an inverted Leica DMi8 microscope. Ki-67 staining was quantified by manually counting the number of Ki-67 positive cells versus the total number of cells (positive for DAPI staining) using ImageJ software. Statistical analysis was performed on raw data using a Repeated Measures ANOVA (with Tukey’s Multiple Comparison Test).

### 4.5. Immunohistochemistry 

Formalin fixed paraffin embedded sections (4 µM) were provided by Derriford hospital, Plymouth, UK (Dr D. Hilton, Cellular and Anatomical Pathology) and were dewaxed, rehydrated and then incubated with KSR1 [(H-70, Santacruz) 1:500] or VPRBP [(DCAF1, proteintech) 1:1000] primary antibodies in Tris-buffered saline with tween (TBST) overnight at 4 ᵒC. Antigen retrieval was performed using citrate buffer [10 mM citric acid (Sigma), pH 6] for KSR1 slides, and Tris-EDTA buffer (20 mM Trizmabase, 1 mM EDTA, pH 9) for VPRBP slides. The Vectastain Universal Elite ABC kit (Vector Laboratories Ltd) was used to visualise proteins, following the manufacturer instructions, and counterstained with haematoxylin (Sigma-Aldrich). The immunohistochemistry was scored in a semi-quantitative manner by a single observer (DH), and the proportion of positive cells estimated. A score of 1–4 was given to each sample corresponding to 0–25%, 25–50%, 50–75% or 75–100%, respectively. Percentage refers to the proportion of cells that are positive for the protein of interest. A Mann–Whitney test or Wilcoxon signed rank test was used to assess statistical significance (*p* < 0.05). 

### 4.6. Lentivirus Production and Infection

MISSION^®^ pLKO.1-puro Non-Mammalian shRNA Control Plasmid DNA (Sigma) was used as a Scramble control for all knockdown experiments. The shDCAF1 lentivirus clone, TRCN0000129909, was purchased from Open Biosystem with a sequence of CCGGGCTGAGAATACTCTTCAAGAACTCGAGTTCTTGAAGAGTATTCT CAGCTTTTTTGGCTGAGAATACTCTTCAAGAA. The human KSR1 sequence, NM _014238.1, with a C-terminal FLAG tag, was cloned into a custom overexpression vector under the CMV promoter (VectorBuilder; Cyagen, Santa Clara, CA, USA). A cleavage linker followed by enhanced green fluorescent protein (EGFP) was used and the plasmid contained ampicillin and puromycin resistance. A control plasmid was used that contained EGFP and puromycin resistance in a similar vector. HEK293FT cells were used to produce lentivirus using a lentiviral reagent mix consisting of pCMV-dR8.91, VSV-G and the hairpin-pLKO.1, or overexpression vector with FuGENE^®^ 6 transfection reagent (in Opti-mem^®^ medium). Supernatant, containing lentiviruses, was collected after 48 h, aliquoted and stored at −80 °C. Cells were grown to at least 70% confluency before infection with lentivirus. Lentivirus was added at a 1:1 ratio with medium supplemented with 16 µg/mL hexadimethrine bromide (Sigma) (cell lines) or protamine sulphate (Sigma) (primary cells) for 24 h. HEK293T cells were split after 4 days puromycin selection to achieve 70% confluency, and then infected with hK1 lentivirus for 48 h followed by cell lysis and FLAG immunoprecipitation. Primary cells were selected with puromycin for a total of seven days, and DCAF1 downregulation was assessed by Western blot. 

### 4.7. Western Blotting 

Primary cells were grown to confluency and lysed in RIPA buffer containing Halt protease and phosphatase inhibitors (1:1000). Protein concentration was determined using the PierceTM bicinchoninic acid Protein Assay Kit (Thermo Fisher Scientific). In total, 20 µg protein was separated on 8% Laemmli SDS-PAGE, and transferred onto polyvinylidene difluoride membrane (Immun-Blot^®^ PVDF membrane, Bio-Rad). Membranes were blocked in a 5% w/v milk (Sigma) and 2% w/v BSA in TBST solution before incubation with primary antibody overnight at 4 °C. Primary antibodies used are listed in Table S1. Secondary HRP-conjugated antibody (Bio-Rad) (1:5000) was added and immunoreactivity detected using the ECL or ECL plus Western Blotting substrate (PierceTM). Membranes were developed by exposure to Amersham Hyperfilm ECL (GE healthcare) or using the PXi gel imaging system (Syngene). Image J was used for quantification of all Western blot bands, using Glyceraldehyde 3-phosphate dehydrogenase (GAPDH) as a loading control. Statistical analysis was performed on the raw densitometry values using Graphpad Prism 5, *p* < 0.05. A Student’s Paired *T* test or a Student’s Unpaired *T* test were used to assess two samples. A one-way ANOVA/Repeated Measures ANOVA was used for more than two samples with Tukey’s Multiple Comparison Test (*p* < 0.05).

## 5. Conclusions

We identified that the simultaneous targeting of CRL4-DCAF1 ubiquitin ligase activity and the Raf/MEK/ERK pathway had additive effects in inhibiting the proliferation and viability of Merlin-deficient meningioma cells. Therefore, MLN3651 treatment, in combination with selumetinib may be a potential future treatment for Merlin-deficient meningioma. 

## Supplementary Materials

The following are available online at https://www.mdpi.com/2072-6694/12/7/1744/s1, Figure S1: (A) Additional immunohistochemical images of normal meninges (200x) and meningioma (200x) tissue stained with DCAF1 antibodies and counterstained with haematoxylin; (B) Additional immunohistochemical images of normal meninges (200x) and meningioma (200x) tissue stained with KSR1 antibodies and counterstained with haematoxylin, Figure S2: (A) Representative immunohistochemical images of normal nerve (NF+/+), traumatic neuroma and Merlin-deficient schwannoma (NF−/−) tissue stained with DCAF1 antibody and counterstained with haematoxylin are shown at 400× magnification (*n* = 5). DCAF1 expression was increased in schwannoma compared to normal nerve and traumatic neuroma, particularly in the nucleus, scale bar—50 µM; (B) The graph shows the proportion of DCAF1 positive cells in five normal nerves, five traumatic neuromas and five schwannoma tissues, where a score of 1 was less than 25% of cells expressing DCAF1, a score of 2 was between 25% and 50%, a score of 3 was between 51% and 75%, and a score of 4 was more than 75%. Schwannoma tissue had significantly more cells expressing DCAF1 than normal nerve, ** *p* < 0.01; (C) Representative Western blot showing Merlin (NF2) expression in human meningeal cells (HMC) and primary meningioma cells (MN), GAPDH—loading control; (D) Densitometry values were calculated from Western blots of DCAF1 and KSR1 protein expression in primary Merlin-deficient meningioma samples relative to the loading control (GAPDH), and plotted. There was a positive correlation between DCAF1 and KSR1 expression; HMC were used as a positive control for the expression of NF2. Meningioma samples with no Merlin expression were used for Merlin-deficient experiments, and assumed to have a Nf2 mutation in at least one allele, Figure S3: Representative Western blots of four primary schwannomas treated with DMSO, 0.3 µM, 0.6 µM or 1 µM MLN3651 for 72 h and probed for LATS2, pYAP and GAPDH. MLN3651 led to an increase in LATS2 and pYAP in all schwannomas, Figure S4: (A) and (B) Meningioma cells were treated with increasing doses of MLN3651, with three technical replicates, and viability was determined after 72 h (*n* = 11). Meningioma cells with an average IC_50_ < 3 µM were plotted in (A) (*n* = 6) and considered to be sensitive to MLN3651 (labelled responsive meningioma), whereas meningioma cells with an average IC_50_ > 7 µM were plotted in (B) (*n* = 5), and labelled as less-responsive meningioma; (C) Human meningeal cells (HMC), a normal meningeal control cell line, were treated with MLN3651, and cell viability was assessed after 72 h (*n* = 3). (D) Merlin-positive grade I meningioma cells were treated with increasing doses of MLN3651, with three technical replicates, and viability was determined after 72 h (*n* = 3), Figure S5: (A) and (B) Merlin-deficient and Merlin-positive grade II meningioma cells were treated with increasing doses of MLN3651 and cell viability was assessed after 72 h (*n* = 6) (B) or 144 h (*n* = 4), Figure S6: (A) Western blots of DCAF1, pERK1/2, ERK1/2 and CYCLIN D1 expression after DCAF1 knockdown in three schwannoma (NF) representative of six replicates. Scramble or DCAF1 lentivirus was added to cells for 24 h followed by a 24-h recovery period and then seven days of puromycin selection before cell lysis; (B) Mean DCAF1, pERK1/2 and CYCLIN D1 expression and SEM in shDCAF1-treated NF cells normalised to the loading control (GAPDH) and relative to Scramble, * *p* < 0.05, ** *p* < 0.01, ns: not significant. GAPDH—loading control, pERK1/2 normalised to total ERK1/2 and GAPDH, Figure S7: Meningioma cells were treated with DMSO, 1 µM Sel, 1 µM MLN3651 or 1 µM Sel and 1 µM MLN3651 for 72 hours and stained with Ki-67 antibody and DAPI. At least three images each of ten replicates were quantified to calculate the percentage of Ki-67 positive cells., ***-*p* < 0.001, ns—not significant, Figure S8: The whole western blot image, Table S1: Antibodies used.
