# Supplementary material for: The Potential of MLN3651 in Combination with Selumetinib as a Treatment for Merlin-Deficient Meningioma
Source: Cancers (Basel). 2020 Jun 30;12(7):1744. doi: 10.3390/cancers12071744 (PMC7407567; doi:10.3390/cancers12071744)
Supplement: Supplementary file 1 [file cancers-12-01744-s001.pdf]

cancers

Article

The Potential of MLN3651 in Combination with Selumetinib as a Treatment for Merlin-Deficient Meningioma

Jade Lyons Rimmer [1], Emanuela Ercolano [1], Daniele Baiz [1], Mahindra Makhija [2], Allison Berger [3], Todd Sells [3], Steve Stroud [3], David Hilton [4], Claire L Adams [1] and C Oliver Hanemann [1,*]

[1] Peninsula Schools of Medicine and Dentistry, Institute of Translational and Stratified Medicine, Plymouth University, Plymouth PL68BU, UK; jade.lyonsrimmer@plymouth.ac.uk (J.L.R.); emanuela.ercolano@plymouth.ac.uk (E.E.); danbaiz73@gmail.com (D.B.); claire.adams@plymouth.ac.uk (C.L.A.)

[2] Takeda International UK, 1 Kingdom Street, London W2 6BD, UK; mahindra.makhija@takeda.com

[3] Millennium Pharmaceuticals, Inc. a wholly owned subsidiary of Takeda Pharmaceutical Company Limited; Cambridge, MA 02139, USA; Allison.Berger@Takeda.com (A.B.); toddsells01@gmail.com (T.S.); steve.stroud@takeda.com (S.S.)

[4] Department of Histopathology, University Hospitals Plymouth NHS Trust, Plymouth, Devon, PL6 8DH, UK; davidhilton@nhs.net

* Correspondence: oliver.hanemann@plymouth.ac.uk; Tel.: +44-1752-437-418

Supplementary

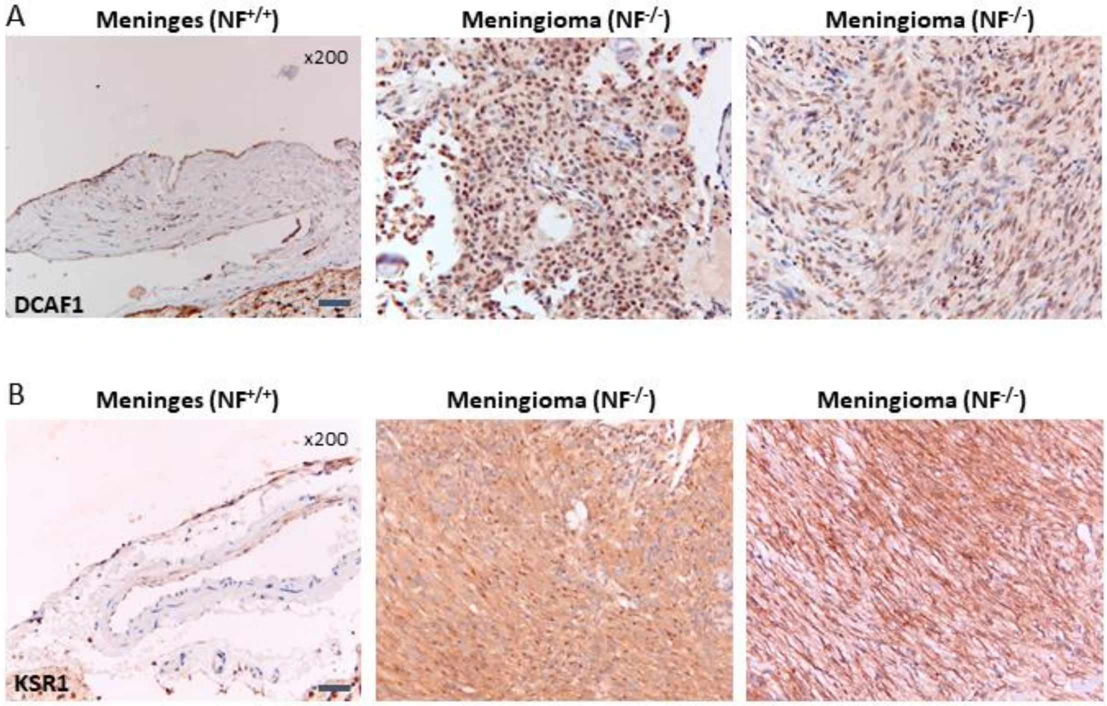

Figure S1. (**A**) Additional immunohistochemical images of normal meninges (200×) and meningioma (200×) tissue stained with DCAF1 antibodies and counterstained with haematoxylin; (**B**) Additional immunohistochemical images of normal meninges (200×) and meningioma (200×) tissue stained with SKR1 antibodies and counterstained with haematoxylin.

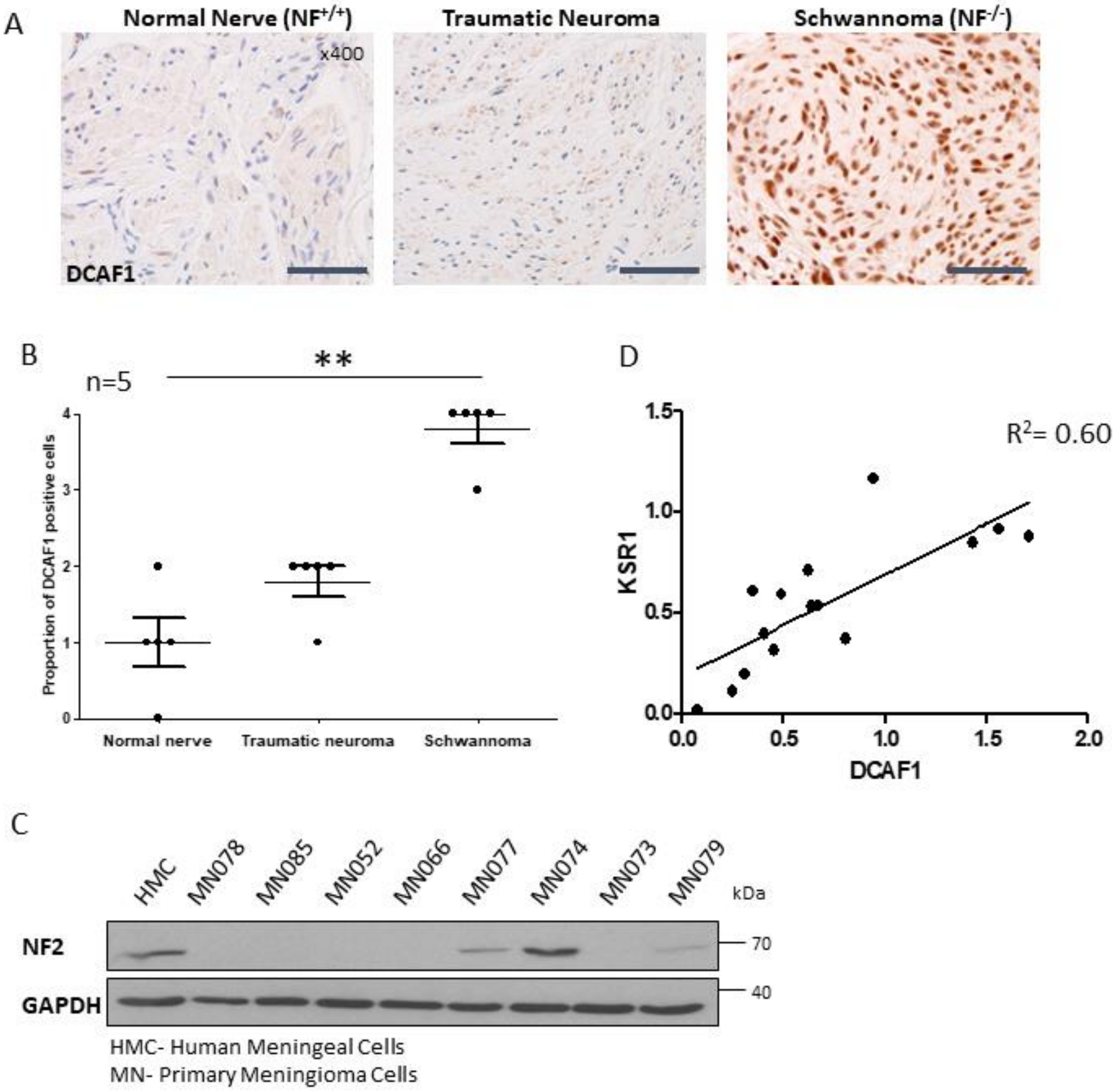

Figure S2. (**A**) Representative immunohistochemical images of normal nerve (NF+/+), traumatic neuroma and Merlin-deficient schwannoma (NF−/−) tissue stained with DCAF1 antibody and counterstained with haematoxylin are shown at 400× magnification ($n = 5$). DCAF1 expression was increased in schwannoma compared to normal nerve and traumatic neuroma, particularly in the nucleus, scale bar—50 µM; (**B**) The graph shows the proportion of DCAF1 positive cells in five normal nerves, five traumatic neuromas and five schwannoma tissues, where a score of 1 was less than 25% of cells expressing DCAF1, a score of 2 was between 25% and 50%, a score of 3 was between 51% and 75%, and a score of 4 was more than 75%. Schwannoma tissue had significantly more cells expressing DCAF1 than normal nerve, ** $p < 0.01$; (**C**) Representative Western blot showing Merlin (NF2) expression in human meningeal cells (HMC) and primary meningioma cells (MN), GAPDH—loading control; (**D**) Densitometry values were calculated from Western blots of DCAF1 and KSR1 protein expression in primary Merlin-deficient meningioma samples relative to the loading control (GAPDH), and plotted. There was a positive correlation between DCAF1 and KSR1 expression; HMC were used as a positive control for the expression of NF2. Meningioma samples with no Merlin expression were used for Merlin-deficient experiments, and assumed to have a Nf2 mutation in at least one allele. The whole western blot image please find in Figure S8.

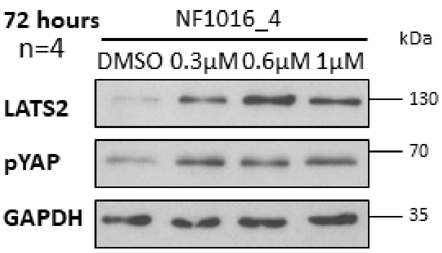

Figure S3. Representative Western blots of four primary schwannomas treated with DMSO, 0.3 µM, 0.6 µM or 1 µM MLN3651 for 72 h and probed for LATS2, pYAP and GAPDH. MLN3651 led to an increase in LATS2 and pYAP in all schwannomas. The whole western blot image please find in Figure S8.

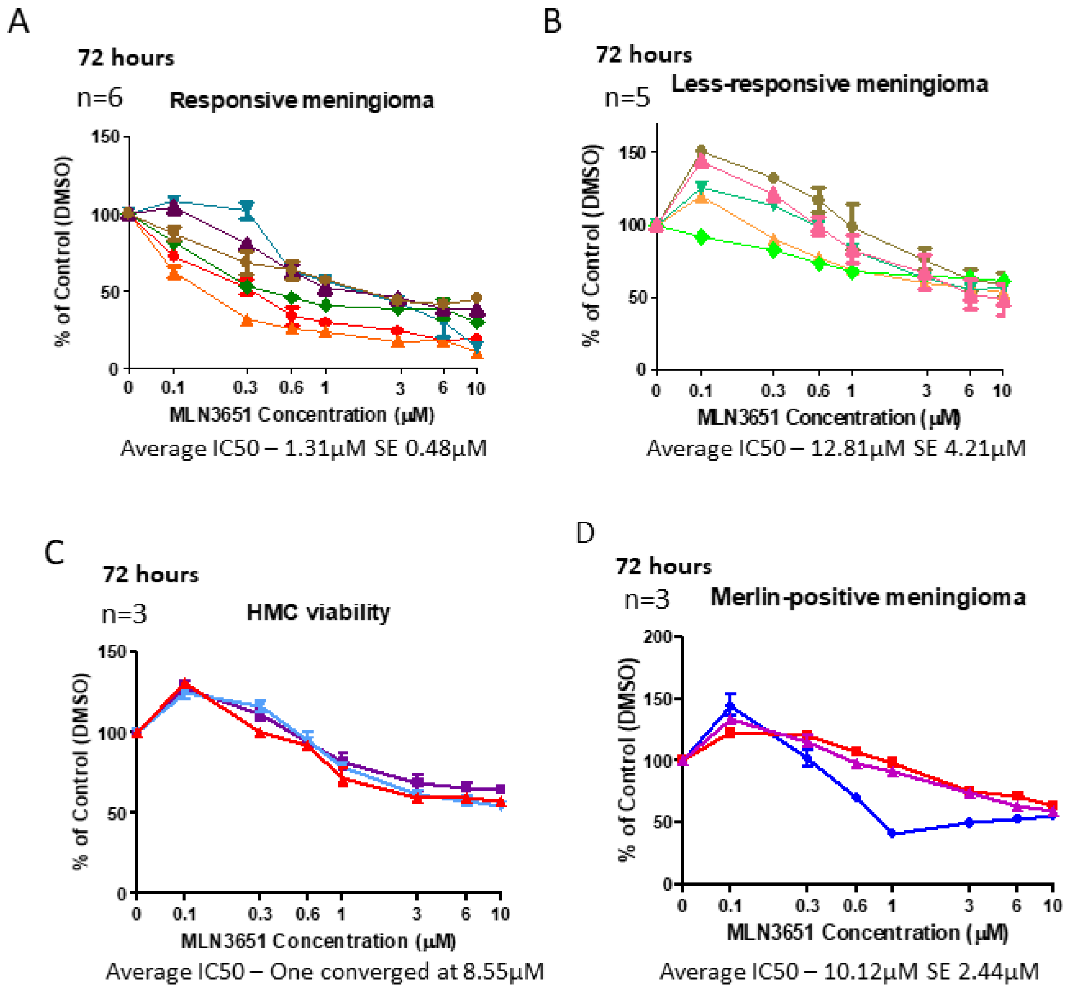

Figure S4. **(A)** and **(B)** Meningioma cells were treated with increasing doses of MLN3651, with three technical replicates, and viability was determined after 72 h (*n* = 11). Meningioma cells with an average IC_{50} < 3 µM were plotted in (**A**) (*n* = 6) and considered to be sensitive to MLN3651 (labelled responsive meningioma), whereas meningioma cells with an average IC_{50} > 7 µM were plotted in (**B**) (*n* = 5), and labelled as less-responsive meningioma; **(C)** Human meningeal cells (HMC), a normal meningeal control cell line, were treated with MLN3651, and cell viability was assessed after 72 h (*n* = 3). (**D**) Merlin-positive grade I meningioma cells were treated with increasing doses of MLN3651, with three technical replicates, and viability was determined after 72 h (*n* = 3). The whole western blot image please find in Figure S8.

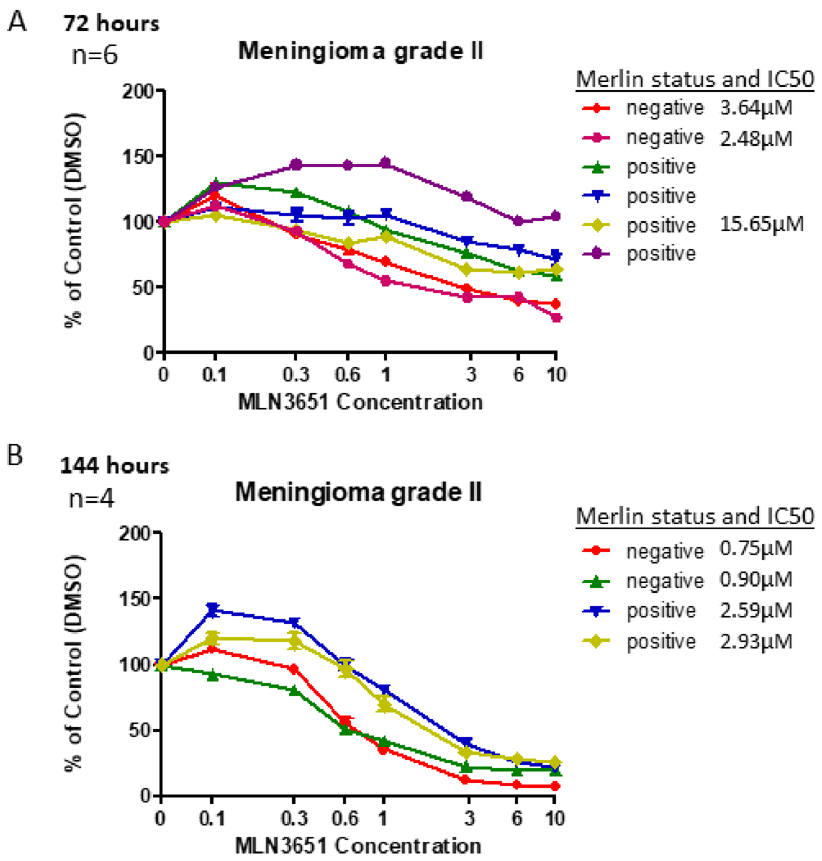

Figure S5. (**A**) and (**B**) Merlin-deficient and Merlin-positive grade II meningioma cells were treated with increasing doses of MLN3651 and cell viability was assessed after 72 h (n = 6) (**B**) or 144 h (n = 4).

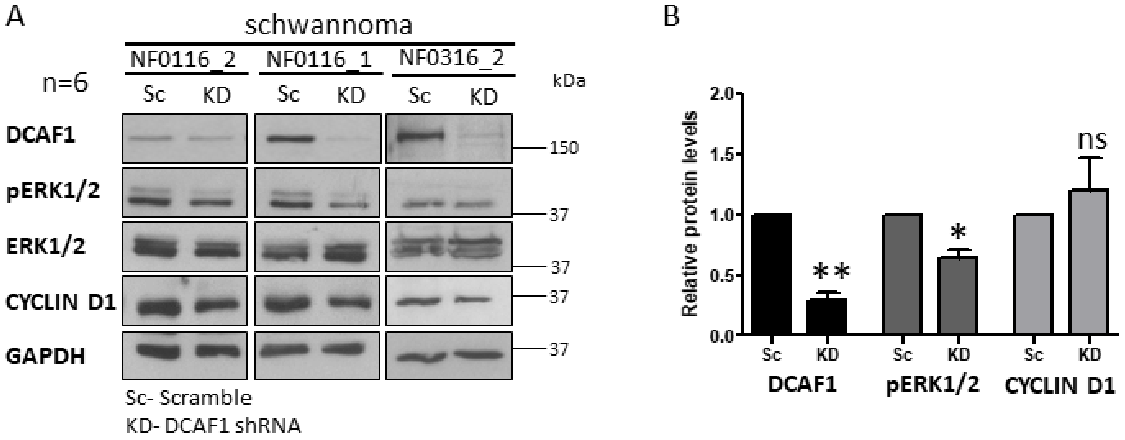

Figure S6. (**A**) Western blots of DCAF1, pERK1/2, ERK1/2 and CYCLIN D1 expression after DCAF1 knockdown in three schwannoma (NF) representative of six replicates. Scramble or DCAF1 lentivirus was added to cells for 24 h followed by a 24-h recovery period and then seven days of puromycin selection before cell lysis; (**B**) Mean DCAF1, pERK1/2 and CYCLIN D1 expression and SEM in shDCAF1-treated NF cells normalised to the loading control (GAPDH) and relative to Scramble, * $p < 0.05$, ** $p < 0.01$, ns: not significant. GAPDH—loading control, pERK1/2 normalised to total ERK1/2 and GAPDH. The whole western blot image please find in Figure S8

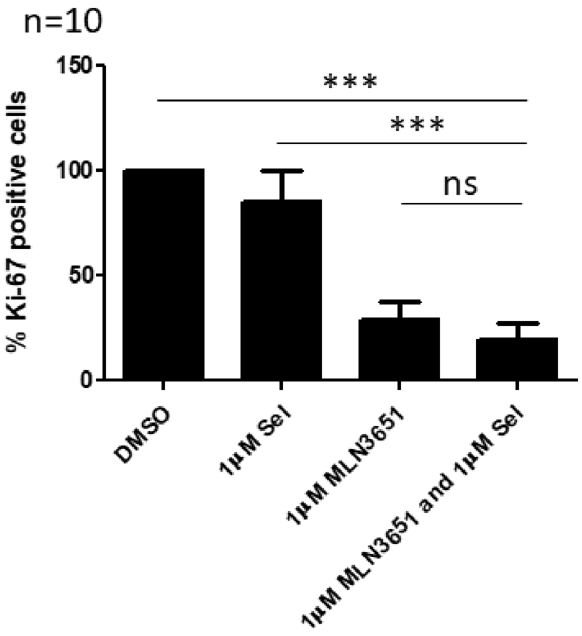

Figure S7: Meningioma cells were treated with DMSO, 1 μM Sel, 1 μM MLN3651 or 1 μM Sel and 1 μM MLN3651 for 72 hours and stained with Ki-67 antibody and DAPI. At least three images each of ten replicates were quantified to calculate the percentage of Ki-67 positive cells., ***-p<0.001, ns—not significant.

Figure 1D

The membrane was cut at 150kDa and 55kDa prior to adding the antibody. KT21 data was not used in the manuscript and Hela was the positive control used in this experiment.

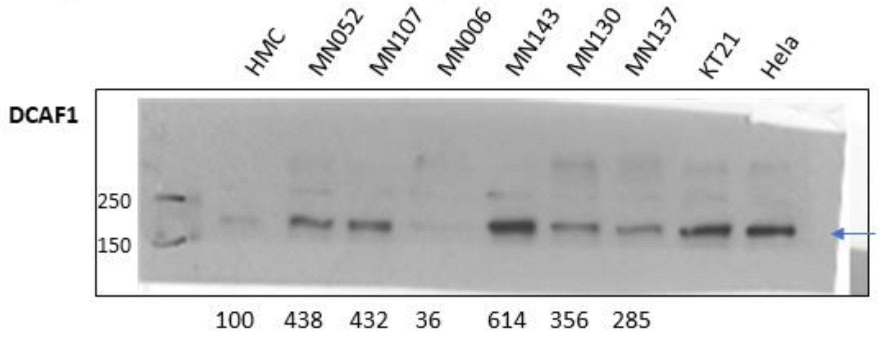

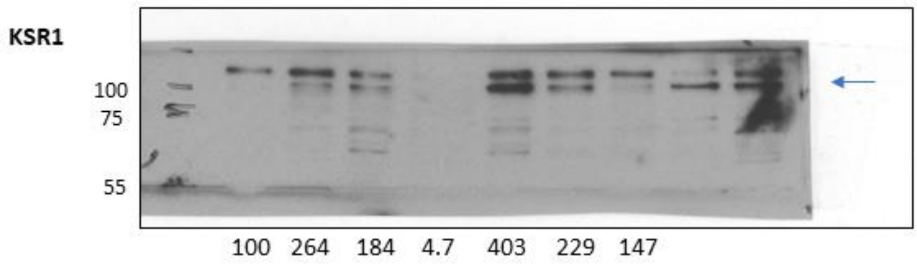

The bands at 42 and 44kDa are total ERK1/2. Total ERK1/2 antibody was added prior to the addition of GAPDH (at 37kDa)

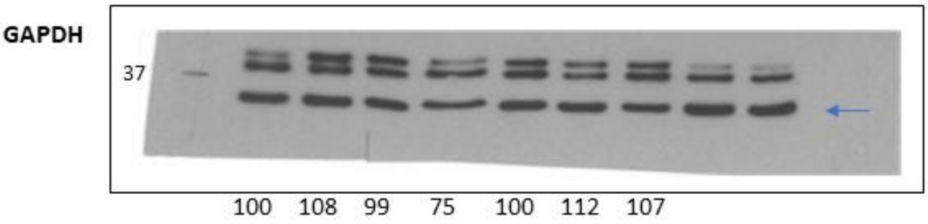

HMC- Human Meningeal Cells
MN- Primary Meningioma Cells

Figure 2C

The blot was stripped after NEDD8 antibody and before adding GAPDH.
HMC was loaded as a positive control

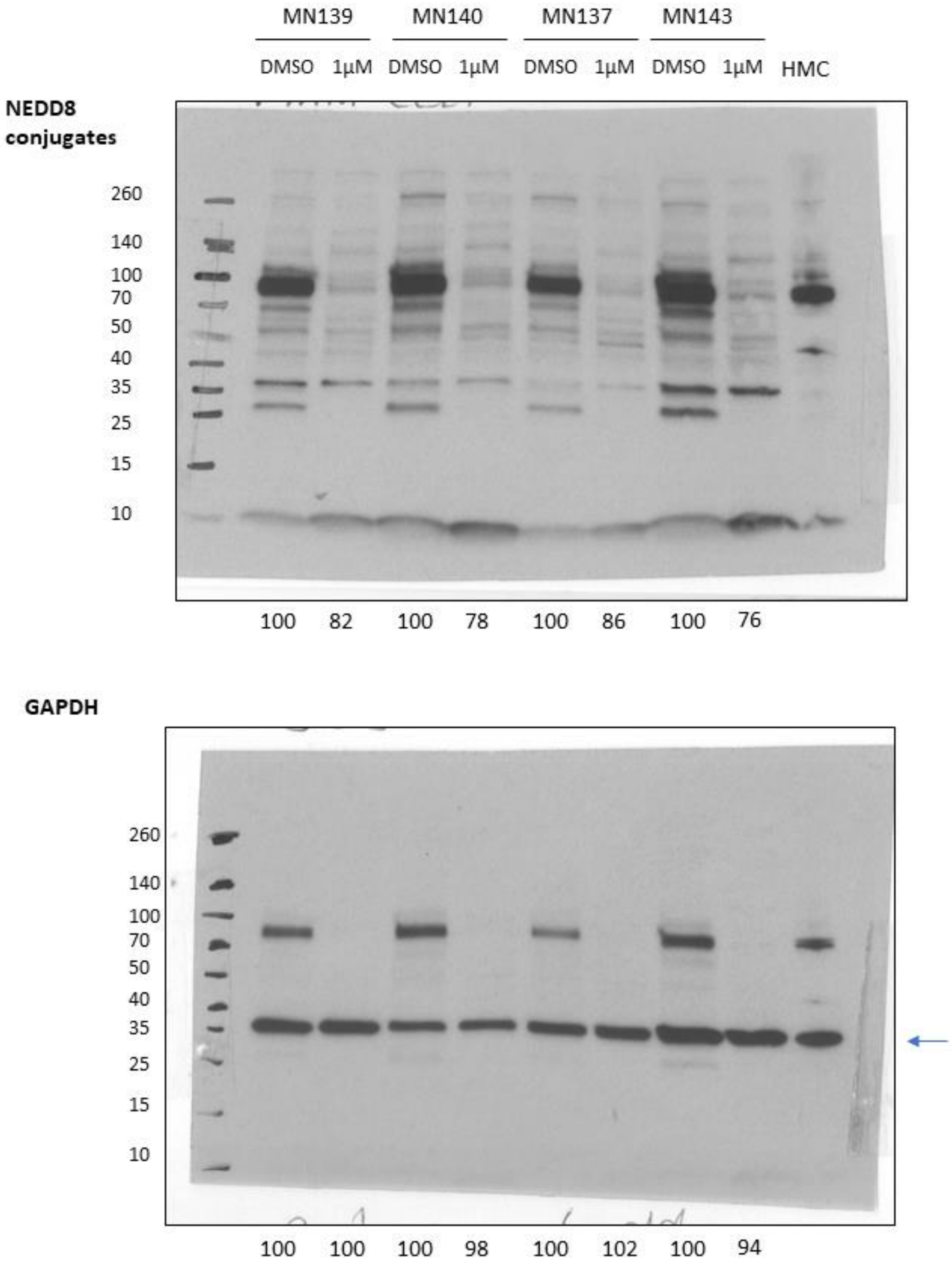

Figure 2D

Two Western blot gels were run with the same lysates and cut at 100kDa and 55kDa. Gel 1 was probed with LATS1, CDT1 and Total ERK1/2 antibodies (then GAPDH). Gel 2 was probed with LATS2, pYAP and pERK1/2 antibodies. The data for CDT1 and pERK1/2 is not shown in the manuscript for this timepoint. Hela was used as a positive control.

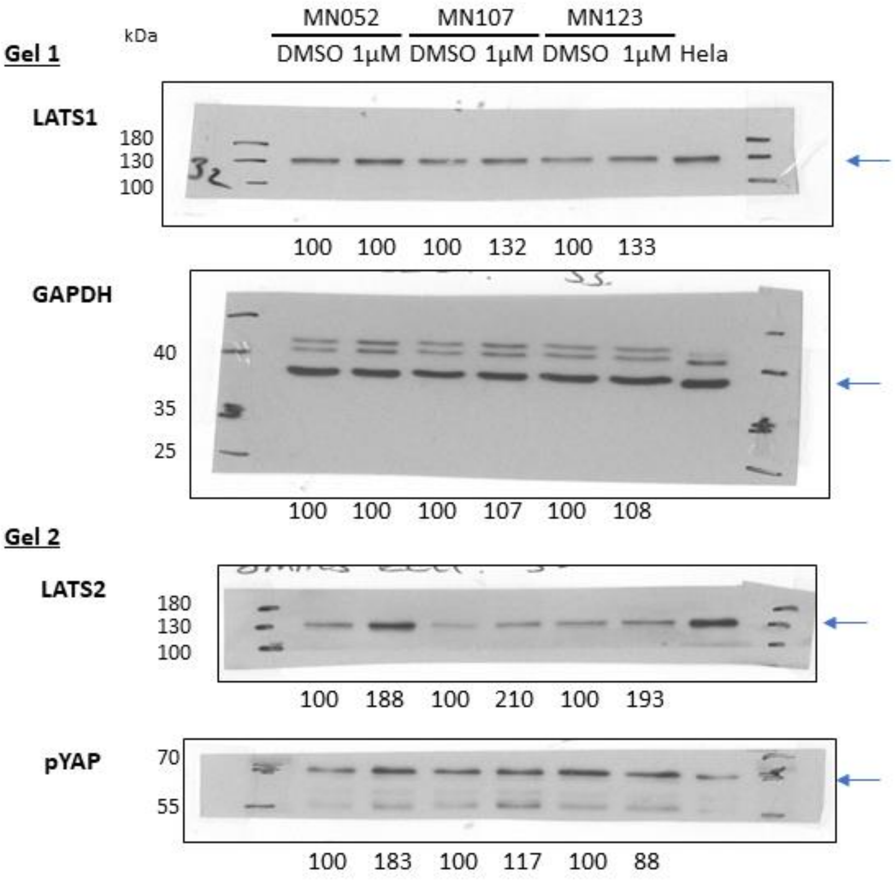

Figure 2E

The blot was cut at 100kDa and 50kDa and probed with Lats2, pYAP and pERK1/2.
The lower blot was stripped and total ERK1/2 was added. Then GAPDH was added.
MN137 and MN073 are additional repeats that were run on the same blots as
MN143 and MN107 and are therefore shown for consistency.

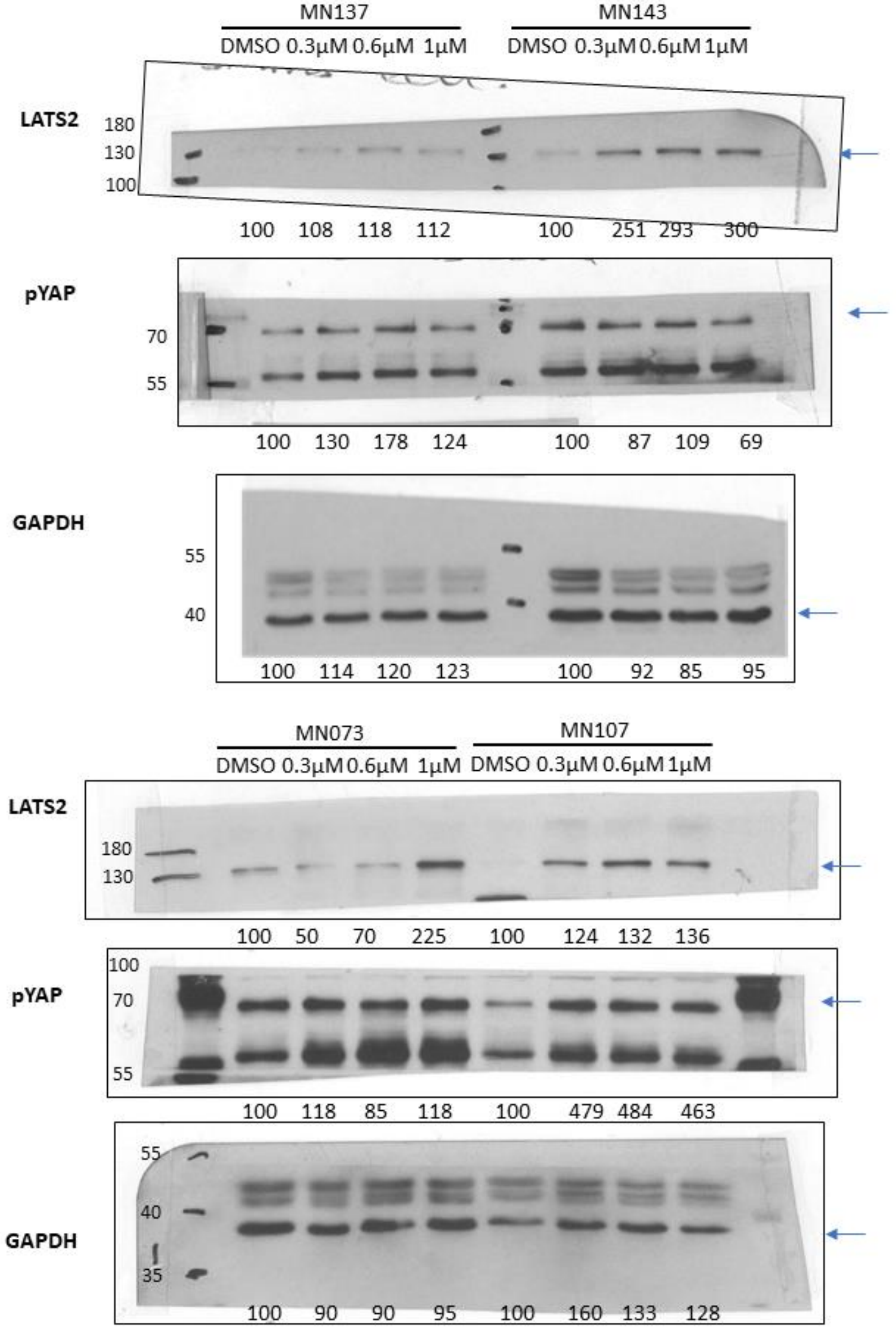

Figure 4A

The blot was cut at 100kDa and 50kDa and probed with Lats2, pYAP and pERK1/2.
The lower blot was stripped and total ERK1/2 was added. Then GAPDH was added.

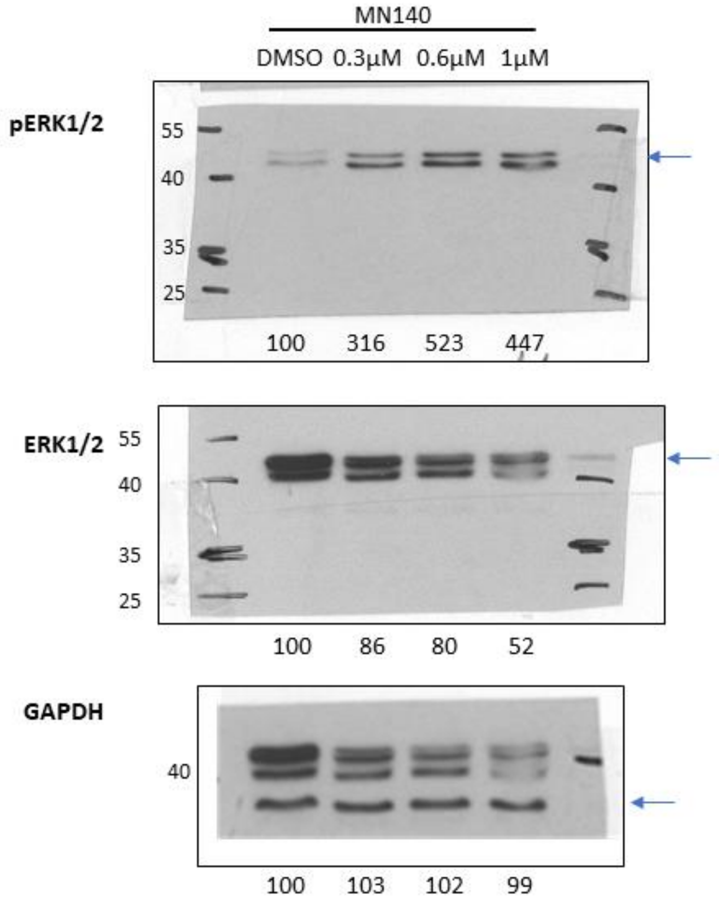

Figure 4B

The blot was cut at 150kDa and 50kDa and probed with DCAF1, KSR1 and pERK1/2. The lower blot was stripped and then GAPDH was added. BenMen-1 data was not shown in the manuscript.

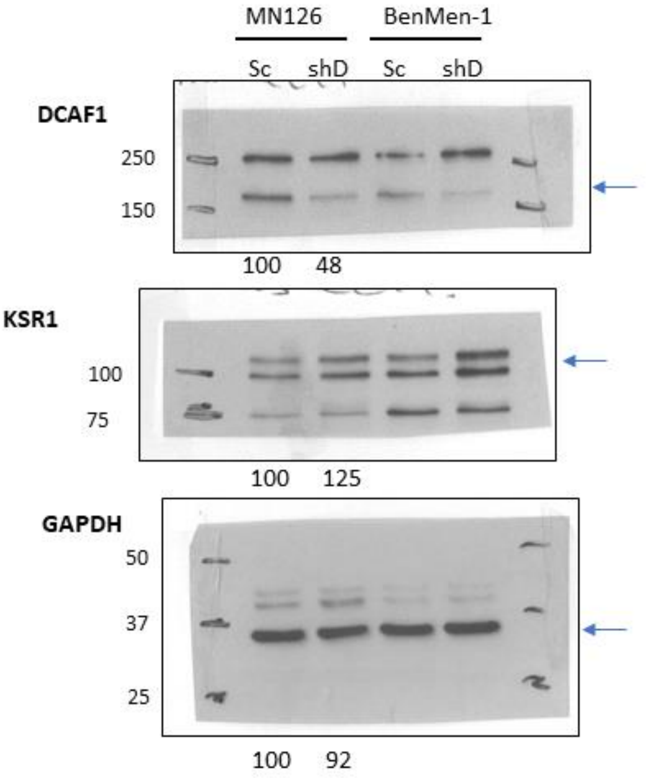

Figure 4C

The blot was cut at 130kDa and 55kDa.

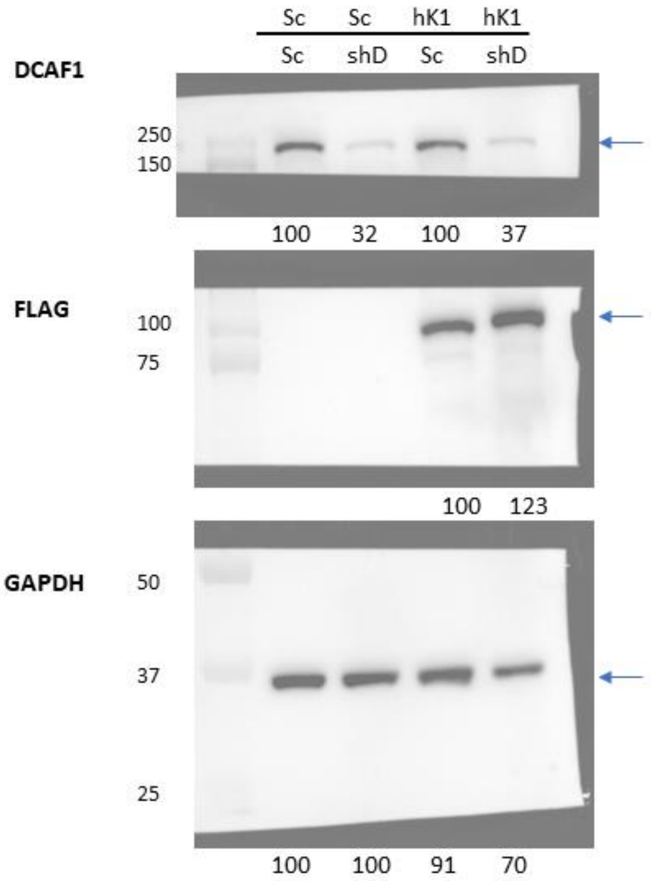

Figure 4D

Four gels were run with the same lysates. Gel 1 was cut at 50kDa and probed with FLAG and ERK1/2. Gel 2 was cut at 50kDa and probed with DCAF1 and pERK1/2. Gel 3 was probed with MEK1/2. Gel 4 probed with c-RAF and pMEK1/2. Longer exposure times are shown that were used for IP samples in the main figure.

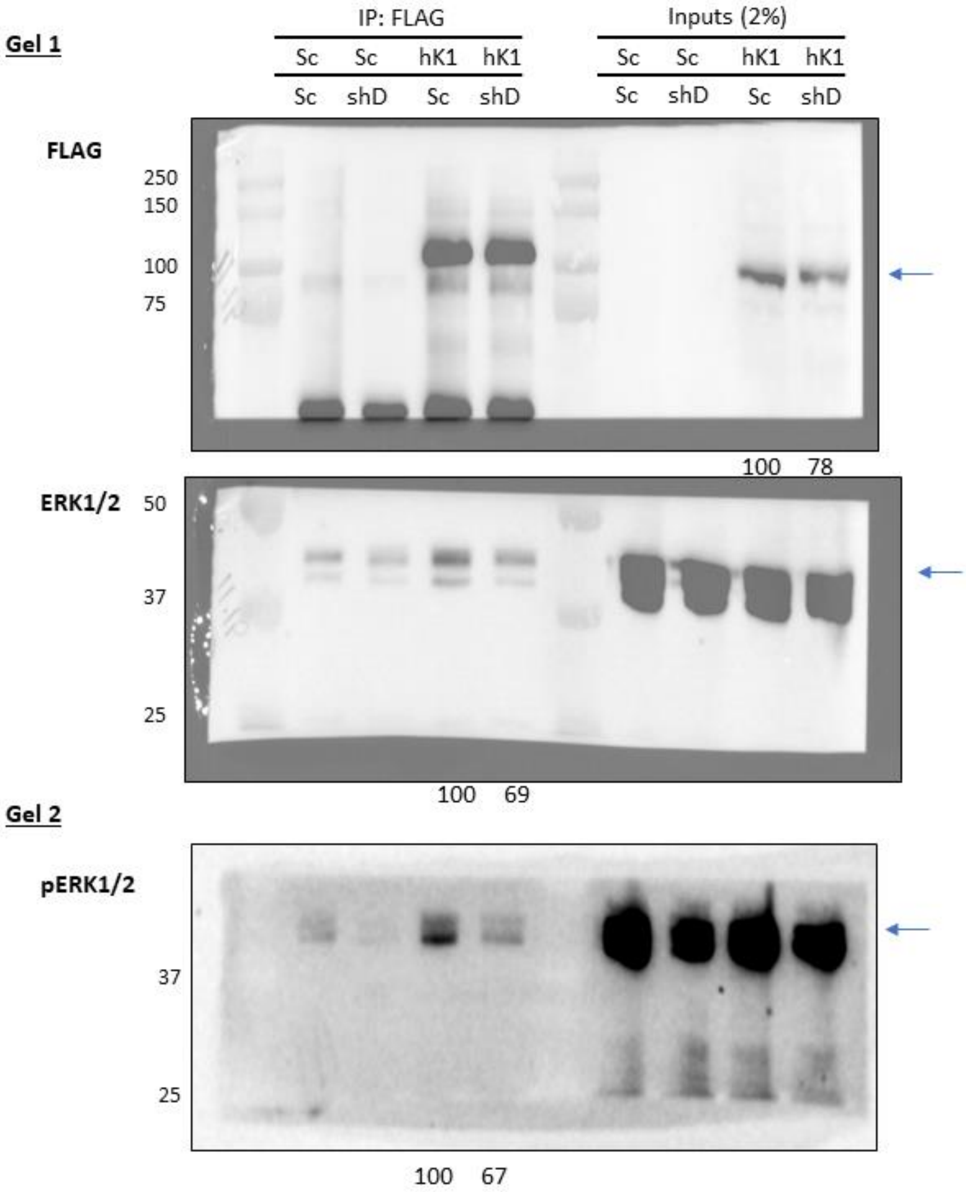

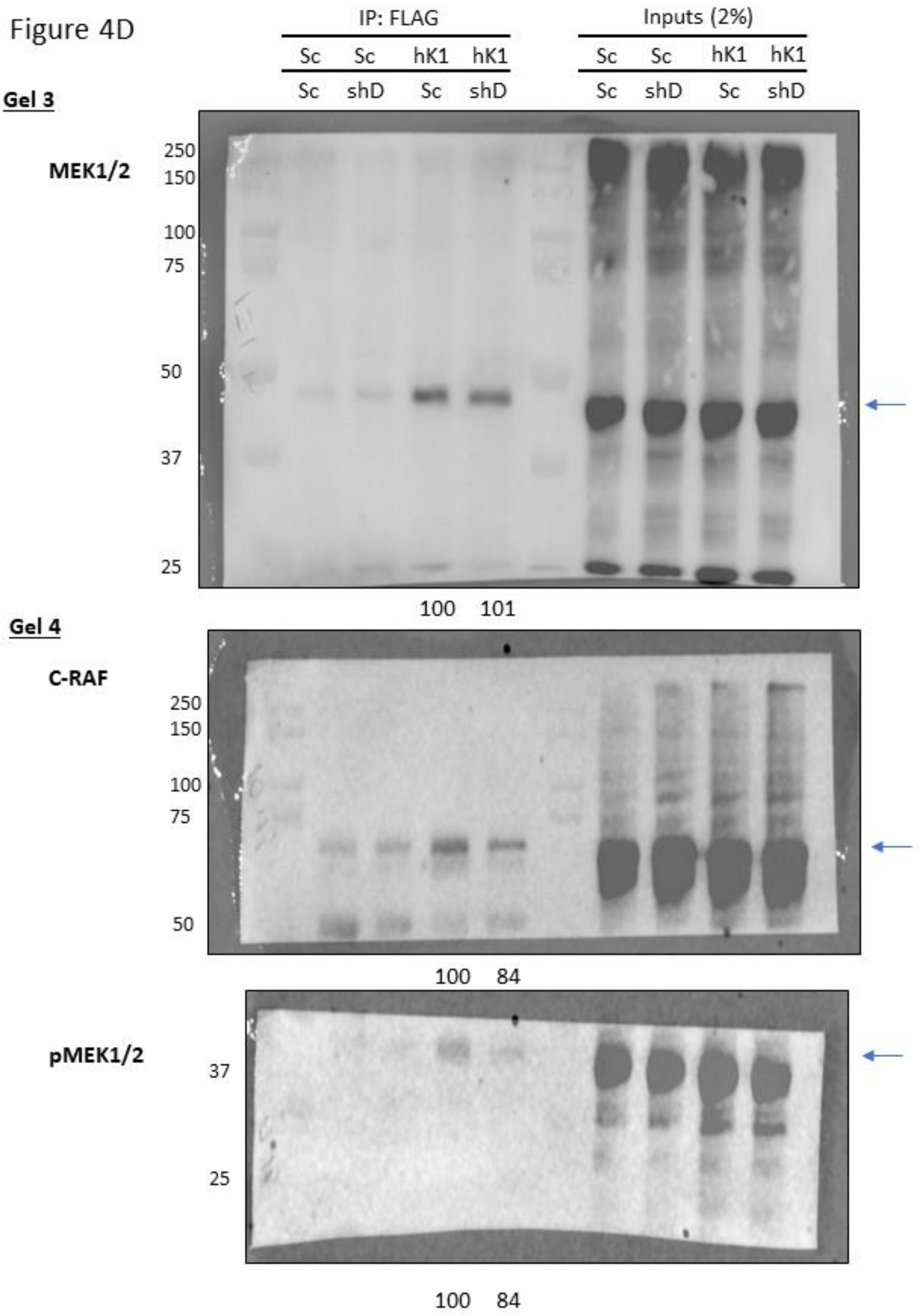
Figure 4D
IP: FLAG
Inputs (2%)
Sc Sc hK1 hK1
Sc shD Sc shD
Sc Sc hK1 hK1
Sc shD Sc shD
Gel 3
MEK1/2
250
150
100
75
50
37
25
100 101
Gel 4
C-RAF
250
150
100
75
50
100 84
pMEK1/2
37
25
100 84

Figure 4D

Shorter exposure times which were used for input samples in the main figure. pERK1/2 was stripped and re-probed with GAPDH.

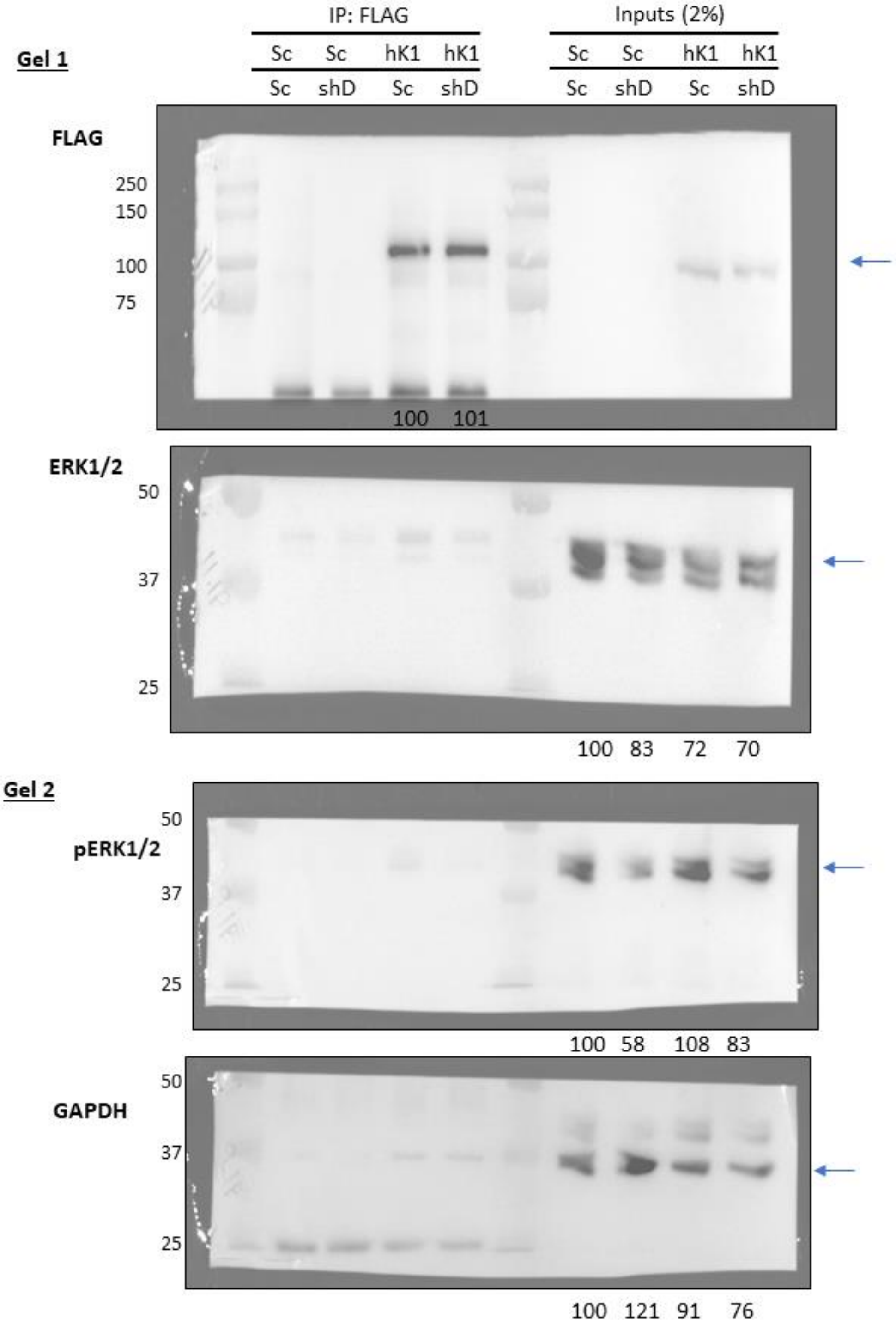

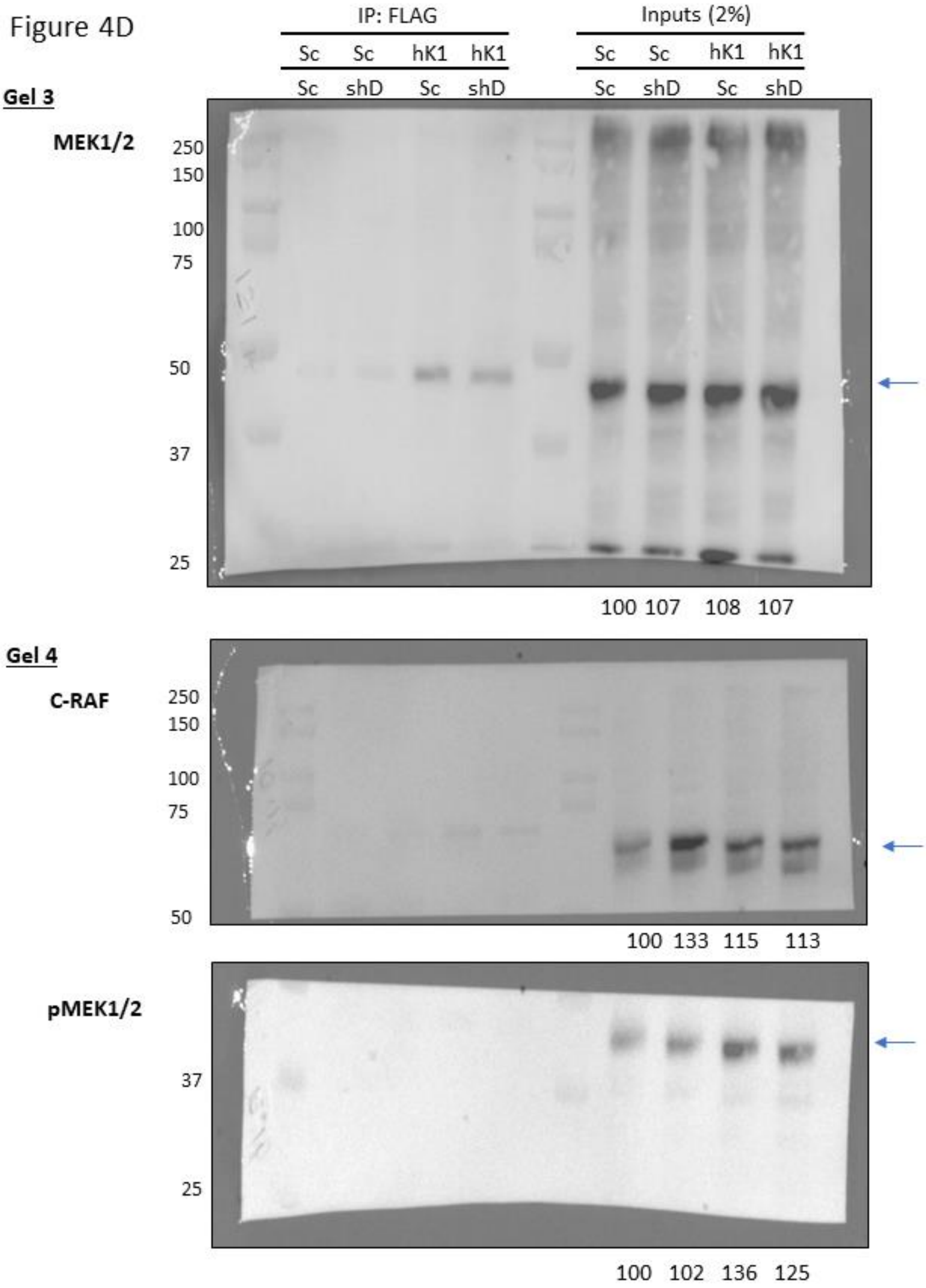
Figure 4D
IP: FLAG
Inputs (2%)
Sc Sc hK1 hK1
Sc shD Sc shD
Sc Sc hK1 hK1
Sc shD Sc shD
Gel 3
MEK1/2
250
150
100
75
50
37
25
100 107 108 107
Gel 4
C-RAF
250
150
100
75
50
100 133 115 113
pMEK1/2
37
25
100 102 136 125

Figure 4E

The blot was cut at 130kDa and 50kDa and probed with DCAF1, KSR1 and pERK1/2.
The lower blot was stripped and then total ERK1/2 was added and then GAPDH.
The lower blot was stripped again and Cyclin D1 was added.

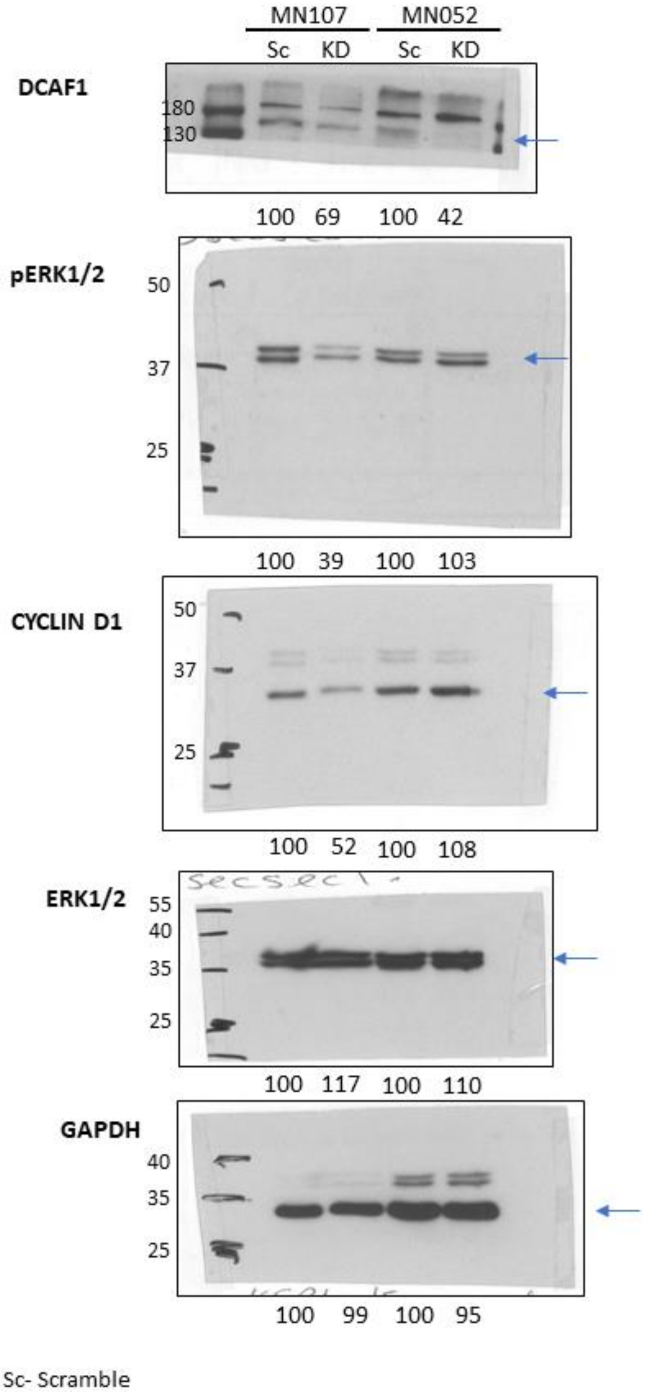

Sc- Scramble
KD- DCAF1 shRNA

Figure 4E

Three blots were run using the same lysates and cut at 130kDa and 50kDa. Gel 1 was probed with pERK1/2, gel 2 was probed with DCAF1, KSR1 and ERK1/2 then GAPDH. Gel 3 was probed with cyclin D1.

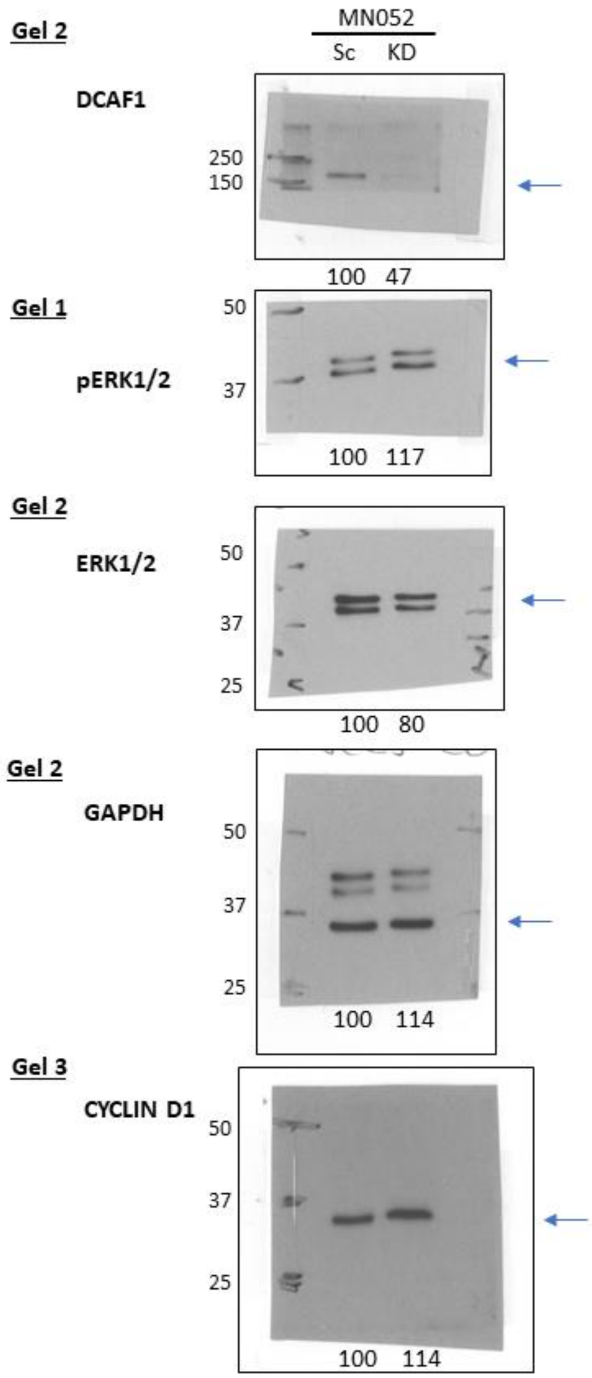

Figure 4E

The blot was cut at 150kDa and 50kDa and probed with DCAF1, KSR1 and pERK1/2. The lower blot was stripped and then total ERK1/2 was added and then GAPDH. BenMen-1 data was not shown in the manuscript.

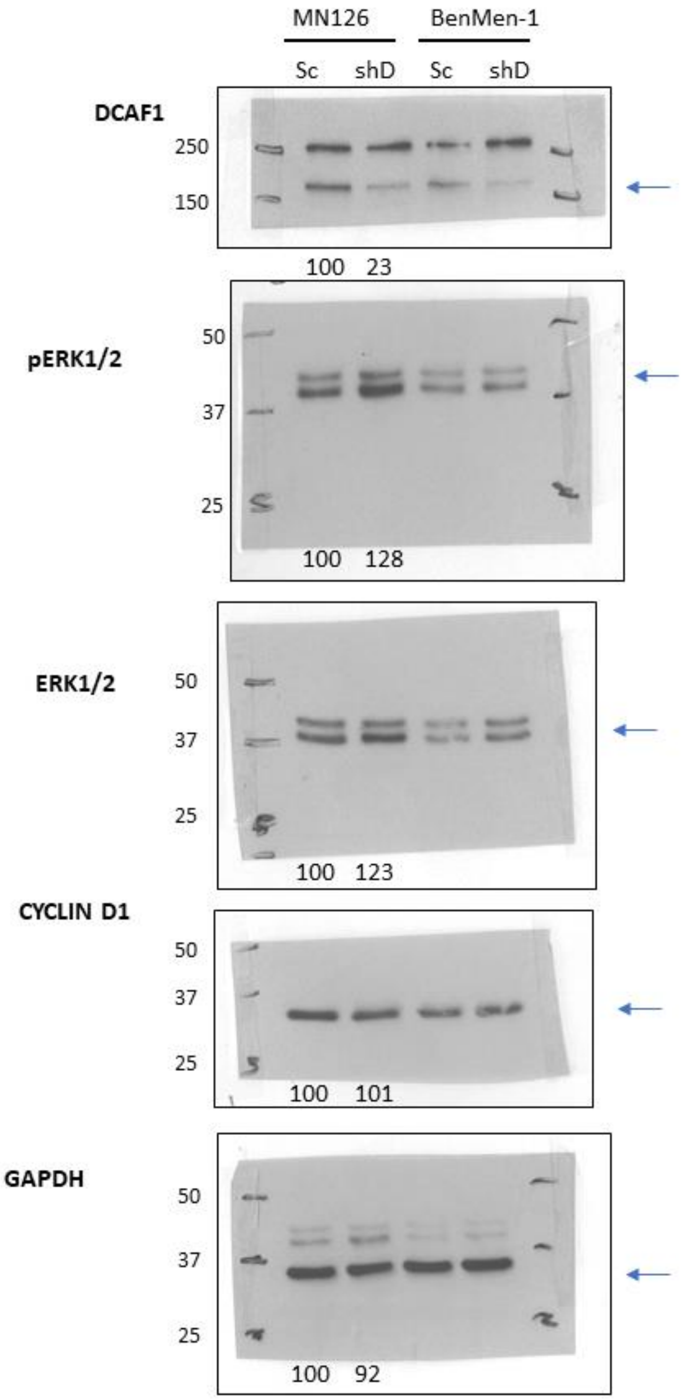

Figure 5A

Two gels were run with the same lysates at cut at 100kDa and 50kDa. Gel 1 was probed with LATS2, CDT1 and Total ERK1/2. The lower blot was then probed with GAPDH. Gel 2 was probed with LATS1, pYAP and pERK1/2. The lower blot was then probed with cyclin D1. .

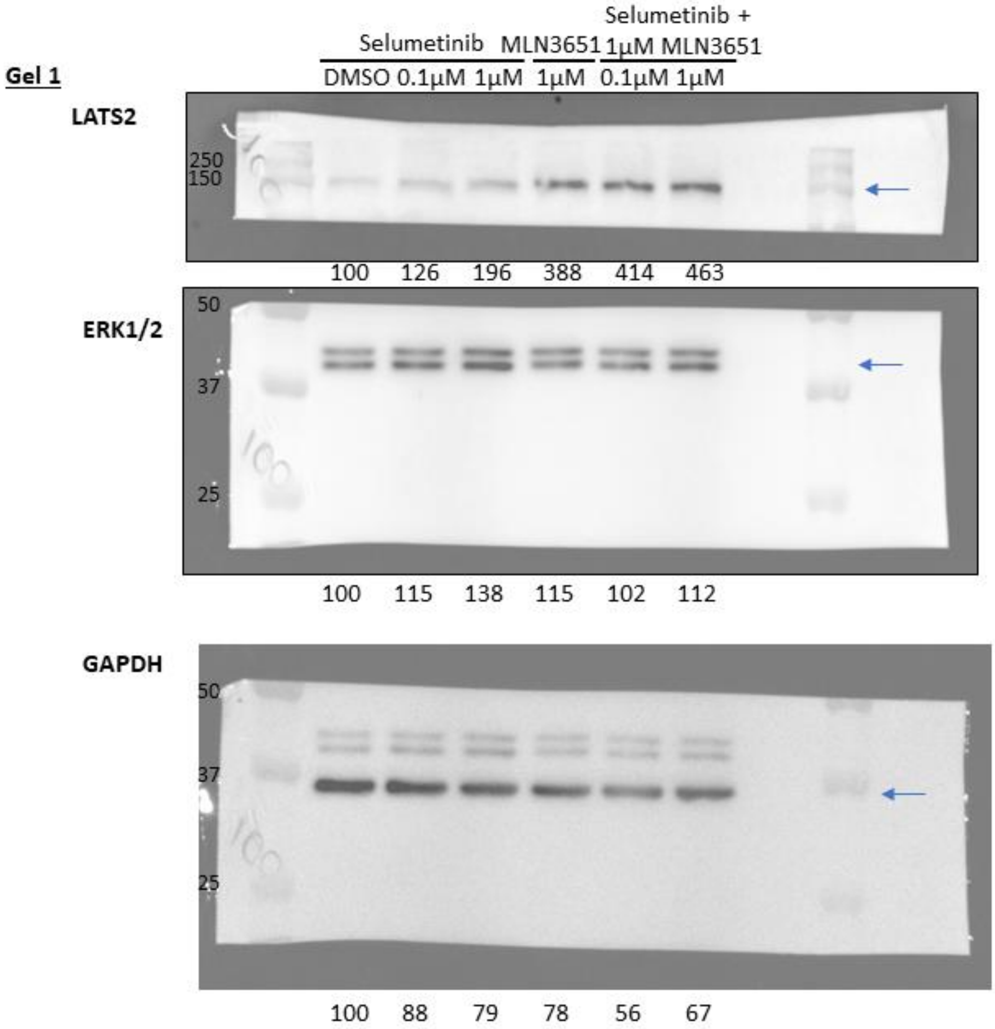

Figure 5A

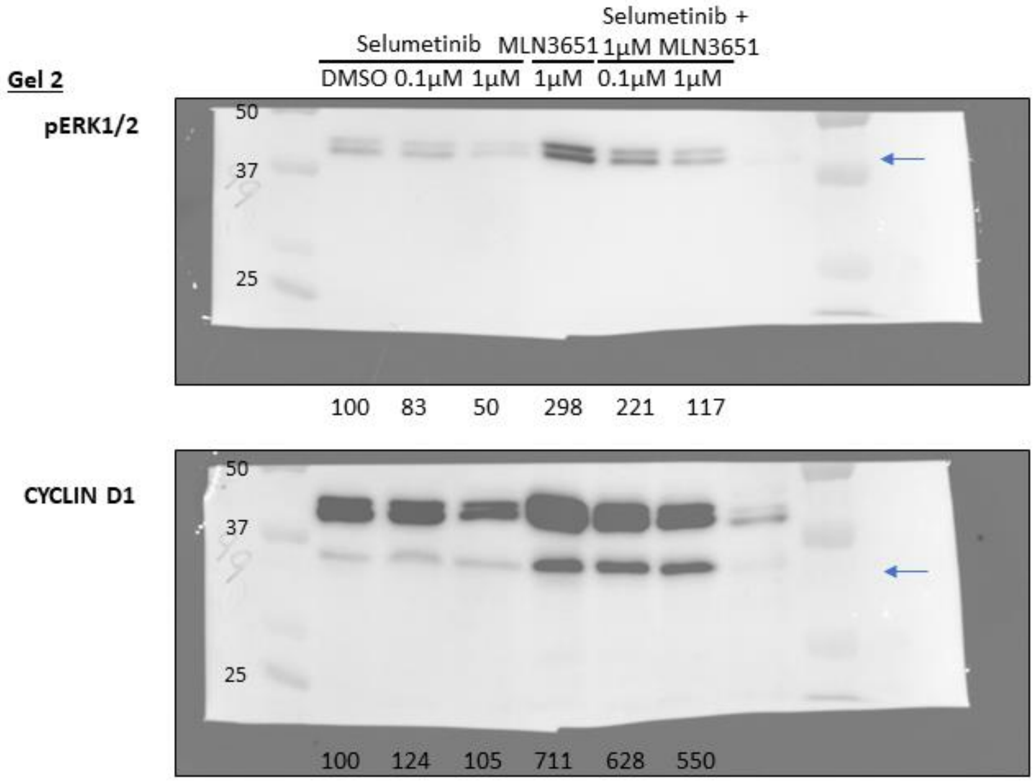
Gel 2
Selumetinib
MLN3651
Selumetinib +
1μM MLN3651
DMSO 0.1μM 1μM 1μM 0.1μM 1μM
pERK1/2
50
37
25
100 83 50 298 221 117
CYCLIN D1
50
37
25
100 124 105 711 628 550

Supplementary Figure 1C

The Western membrane was cut at 40kDa prior to adding primary antibodies. No quantification necessary as simply determining if the samples are Merlin-positive or Merlin-negative

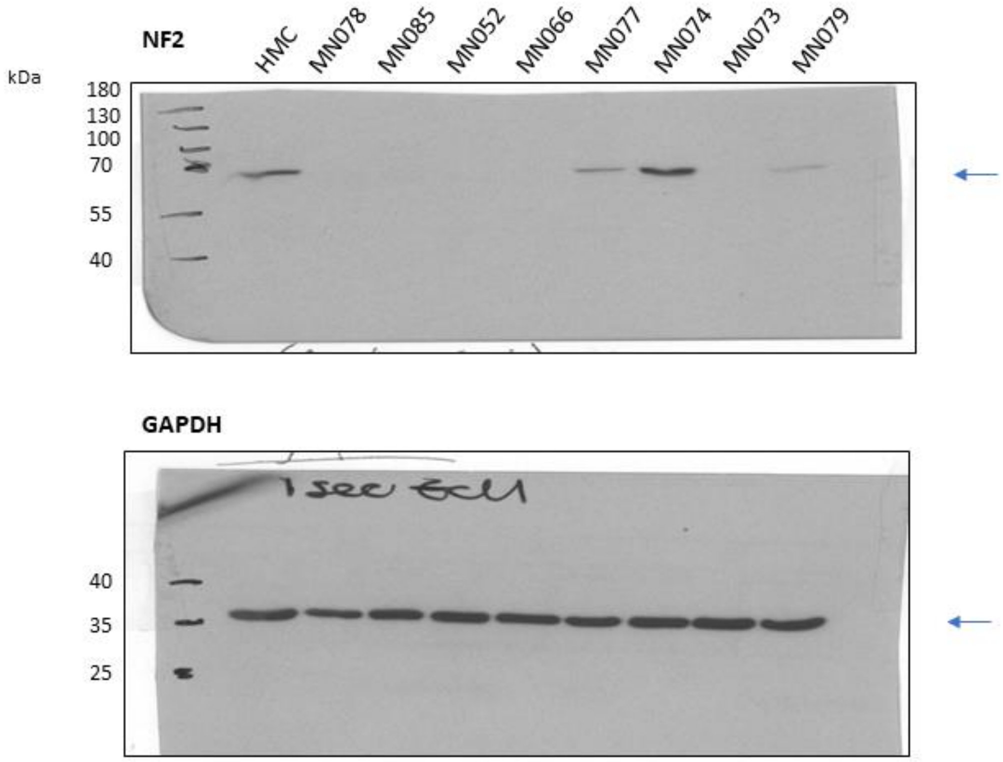

HMC- Human Meningeal Cells
MN- Primary Meningioma Cells

Supplementary Figure 2A

The Western membrane was cut at 100kDa and 40kDa and probed with LATS2, pYAP and GAPDH.

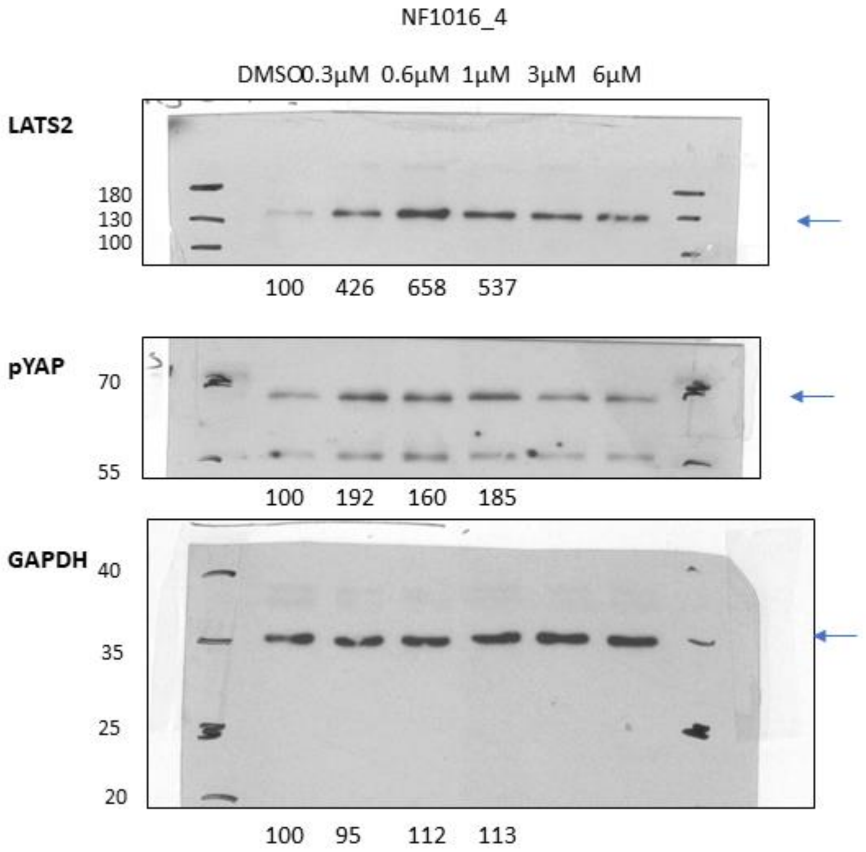

Supplementary Figure 5A

Two blots were run using the same lysates and cut at 75kDa and gel 1 was probed with DCAF1 and pERK1/2 then Cyclin D1. Gel 2 was probed with KSR1 and total ERK1/2 and then GAPDH.

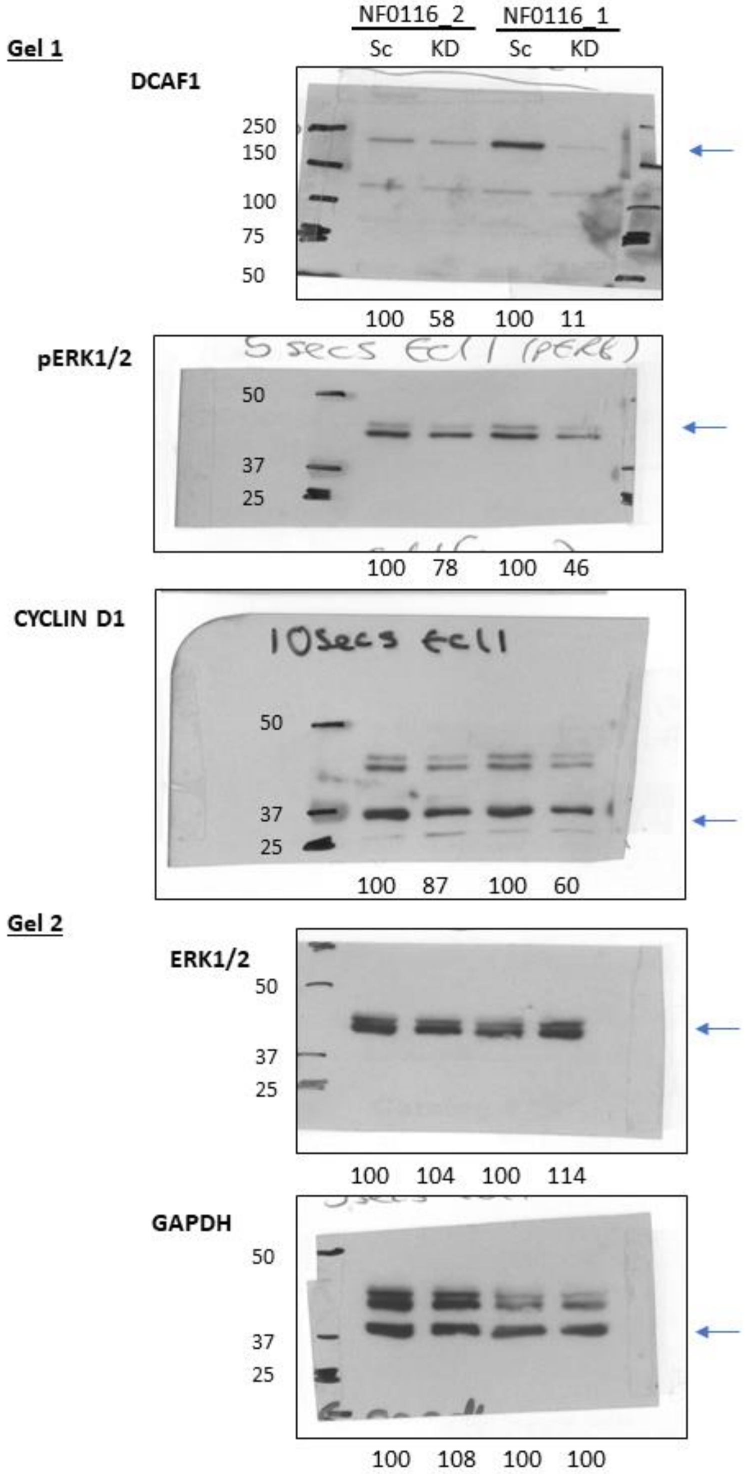

Supplementary Figure 5A

Two blots were run using the same lysates and gel 1 was probed with DCAF1. gel 2 was cut at 50kDa and probed with pERK1/2, stripped and then probed with total ERK1/2 then Cyclin D1 and stripped again then GAPDH was added.

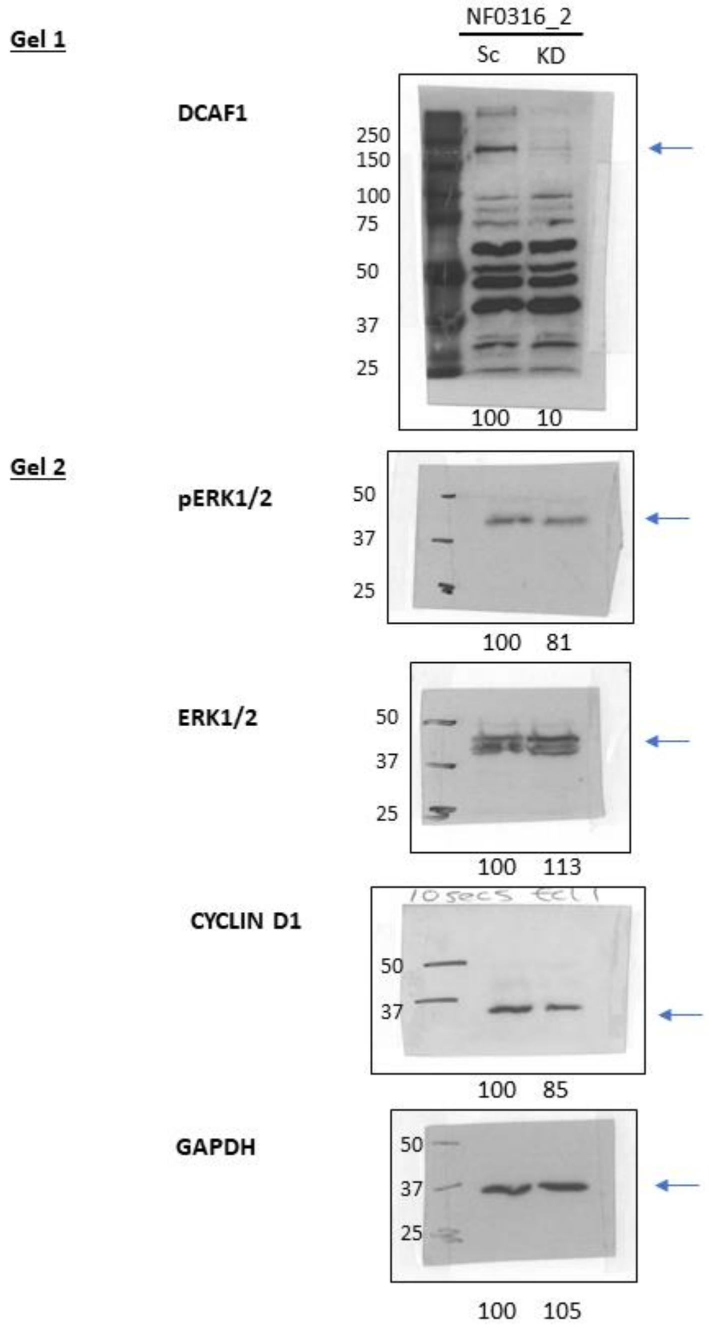

Figure S8. The whole western blot image.

Table S1. Antibodies used.

Antibody	Type	Clone	Company	Catalog	Dilution
C-RAF	Rabbit poyclonal		CST	9422	1:1000
CYCLIN D1	Rabbit poyclonal		CST	2922	1:1000
DDB1	Rabbit poyclonal		Bethyl	A300-462A	1:1000
ERK1/2	Rabbit poyclonal		CST	9102	1:1000
FLAG	Mouse monoclonal	M2	Sigma	F1804	1:5000
GAPDH	Mouse monoclonal	6C5	Millipore	MAB374	1:20000
KSR1	Rabbit poyclonal		CST	4640	1:500
KSR1 H-70	Rabbit poyclonal	H-70	Santa Cruz Biotechnology	Sc-25416	1:500
LATS2	Rabbit poyclonal	D83D6	CST	5888	1:1000
MEK1/2	Rabbit poyclonal		CST	9122	1:1000
NEDD8	Rabbit poyclonal		CST	2745	1:1000
p-ERK (Y202/Y204)	Rabbit poyclonal		CST	9101	1:2000
p-MEK1/2 (S217/S221)	Rabbit poyclonal	41G9	CST	9154	1:1000
p-YAP (S127)	Rabbit poyclonal		CST	4911	1:1000
VPRBP (DCAF1)	Rabbit poyclonal		Proteintech	11612-1-AP	1:1000
